# The 22-item Positive Health questionnaire (PH22) is reliable and applicable among different (Dutch) hospital populations: A test-retest study design

**DOI:** 10.1371/journal.pone.0356368

**Published:** 2026-08-20

**Authors:** Lenny M. W. Nahar - van Venrooij, Babette C. van der Zwaard

**Affiliations:** 1 Jeroen Bosch Academy Research, Jeroen Bosch Hospital’s, Hertogenbosch, the Netherlands; 2 Tranzo Scientific Centre for Care and Wellbeing, Tilburg University, Tilburg, the Netherlands; School of Nursing Sao Joao de Deus, Evora University, PORTUGAL

## Abstract

**Background:**

There is a growing adoption in (healthcare) organizations of Positive Health, a new theoretical concept of health moving from a disease-oriented perspective toward a more holistic understanding of health. Alongside this, there is a growing need to make the impact of working according to this concept measurable. The validated 22-item Positive Health questionnaire (PH22) has good potential for this purpose. However, information about the test-retest reliability of the PH22 is lacking. Also, its performance in patient populations is unknown.

**Objectives:**

In addition to the previous validation study in a general population, this study aimed, in line with international guidelines, to evaluate the (test-retest) reliability of the PH22, and its performance among two different hospital populations. Additionally, its discriminative ability was examined by assessing PH22 scores across subgroups defined by educational level and level of (chronic) disease.

**Methods:**

Participants were recruited from a general hospital population and a lifestyle outpatient population with overweight. Using a test–retest design, the measurement properties: internal consistency, agreement (intraclass correlation coefficient (ICC), measurement error (ME), smallest detectable change (SDC)) and model fit indices were examined using Confirmatory Factor Analysis (CFI, RMSEA, SRMR).

**Results:**

Included were 360 and 139 patients, respectively. Of whom 247 and 121 also completed the retest. Internal consistency of the PH22 was good, except for the dimension Future perspective (Cronbach’s α < 0.7). Agreement was good (ICC > 0.7), except for the dimension Future perspective among the general hospital population (ICC = 0.63, ME = 2.8 (7.3%), SDC = 7.8 (26%)). Overall, the PH22 model fit was acceptable, but for the Lifestyle outpatient population only the RSMEA index was sufficient. As expected on forehand, the PH22 scores were higher among those higher educated and lower for those with a chronic disease or higher healthcare use.

**Conclusion:**

The PH22 appears to be a reliable and applicable instrument for measuring Positive Health among general hospital populations. Caution should be taken among more specific (patients) groups, especially for the dimension Future perspective. Future research should focus on the clinical relevance and interpretation of different PH22 scores.

## Introduction

There is a growing adoption of the concept of Positive Health in organizations both within and beyond the healthcare sector [1–3]. In 2011, Huber et al. [4] introduced this dynamic and broad concept of health, defining health as the ability to adapt to physical, emotional, and social challenges in life and to maintain self-management. This new concept of health moved away from a disease-oriented perspective toward a more holistic understanding of health. Subsequently, in 2016, this theoretical concept was further operationalized into a patient-centered concept of health comprising six dimensions: Bodily functions, Mental well-being, Meaningfulness, Quality of life, Social participation, and Daily functioning, called *Positive Health* (PH) [5].

Alongside the increasing adoption of the PH concept, there is a growing need to make the impact of working according to this concept measurable [3,6]. In the Netherlands, three instruments have been developed to assess self-reported Positive Health among a general (Dutch) population: the 17-item (PH-17) [7,8], the 42-item (PH42) [9,10], and the 22-item Positive Health questionnaire (PH22) [9,11]. All of these measurement instruments originate from the original 42-item dialogue tool My Positive Health, which was derived from the patient-centered operationalization of health proposed by Huber et al.[5].

The *My Positive Health* (MPH) dialogue tool was not developed, nor is it suitable, as a measurement instrument. Its primary purpose is to facilitate a broad conversation about health and to stimulate self-reflection [3,12]. The current version of the dialogue tool consists of 44 items, each scored on an 11-point scale ranging from 0 (“completely disagree”) to 10 (“completely agree”), grouped across the six dimensions of Positive Health. MPH is visualized using a spiderweb format. In contrast, the PH-17 and PH22 instruments (and its precursor the PH42), were explicitly developed for measurement purposes. These instruments differ from the dialogue tool in both item composition and dimensional structure.

The PH22 seems to have the best potential for measuring PH. All three instruments demonstrated structural validity and showed sufficient model fit through cross-validation in a general Dutch population [7,9,11]. However, the internal consistency of the PH-17 and PH42 was not optimal. This was reflected in excessively high internal correlations within several dimensions (Cronbach’s α > 0.9), indicating item redundancy. This redundancy resulted from overlap between items and, in the case of the PH-17, from limited content breadth due to the item selection method employed [8]. To address these limitations, the PH22 was developed through substantive expert discussions informed by the outcomes of extensive statistical analyses [11]. Content, structural validity and internal consistency of the PH22 were judged to be good. The PH22 consists of 22 items and comprises four dimensions: Physical fitness, Satisfaction with self, others and life, Daily self-management, and Future perspective.

The PH22 was initially developed based on a representative cohort of the general population [11]. To meaningfully use PH22 scores for assessing differences between groups and changes over time in order to evaluate the PH approach or patient-centered care, it is essential to quantify the measurement error of the instrument to ensure correct interpretation [13]. Moreover, for the application of the PH22 in hospital settings, it is important to expand assessment of its measurement properties for patient populations beyond the general public. Currently, evidence regarding its test-retest reliability or performance of the PH22 in such populations is lacking. Therefore, this study aimed, in line with international guidelines [13], to evaluate the reliability of the PH22, its responsiveness and performance, including internal consistency, measurement error, smallest detectable change and its model fit, in both a general and a more specific hospital population, using a test–retest design. Additionally, its discriminative ability was further examined by assessing on forehand expected differences in Positive Health scores across subgroups defined by educational level and level of (chronic) disease.

## Methods

### Design and setting

A cross-sectional study including test-retest design was performed among two hospital populations. This research was done at the Jeroen Bosch Hospital (JBH), a teaching hospital with 630 clinical beds located in the south of the Netherlands.

### Participants

The first population was a **general hospital population**. In accordance with standard procedures for administering patient satisfaction surveys by the Department of Communication patients were approached. Included were adult patients (≥18y) who visited the JBH outpatient clinic or were discharged from the hospital, the day before. Excluded were: patients having memory problems or dementia, and patients who registered to prefer post by mail, or not willing to receive surveys or share their data for scientific purposes. Also, patients that already received a patient satisfaction survey in the past 12 months, were excluded. The first consecutive 2000 eligible patients were invited by email during last week of May 2024 (each working day) to send their email address to the researcher if they were interested to participate. If interested, they received an invitation to participate with an electronic link to the PH22 questionnaire using the Electronic Self-service System (ESS) of the Scientific Office. The recruitment period started 30/05/2024 up to 13/06/2024. After seven days the ESS sent the second PH22 questionnaire (retest) (08/06/2024–22/6/2024). The request for the retest was only sent if the first PH22 (test) was completed, with a minimum of four and a maximum of 14 days between the test and retest.

The second population was a population with overweight visiting the hospital Lifestyle outpatient clinic, further called the **lifestyle outpatient population**. At the Lifestyle outpatient clinic conversations using the Positive Health approach and its dialogue tool are applied. This outpatient clinic was one of the first departments in this hospital using this Positive Health approach with also potential to be adopted by other hospitals. Therefore, the PH22 was also studied among this specific population. Included were Dutch speaking adult patients (≥18y) with BMI > 25 referred to the Lifestyle outpatient clinic by their JBH medical or nursing specialist (see S1 Appendix S1 for JBH referral criteria). After planning their first appointment, they receive the brochure *How are you?*, a product from the Institute for Positive Health, per post together with the PH22 questionnaire about one week before their appointment. Accordingly, patients are asked to read the brochure and complete the questionnaire at home, closely after receiving it, in preparation for their visit. At the Lifestyle outpatient clinic they were informed about the research project and asked if they were willing to participate in the test-retest study. If so, they completed the PH22 again at location just prior to their first appointment (retest). Patients were recruited over one year expecting 200 visitors starting 04/01/2024 (up to 30/09/2024).

### Ethical approval and consent to participate

Both studies were conducted in accordance with current public regulations, laws, and the principles of the Declaration of Helsinki.. Informed consent was given by each participant to be included. Informed consent was obtained written for the **general hospital study population** and verbal for the **lifestyle outpatient population** (which was documented by their healthcare provider at the electronic patient file). The Medical Ethics Committee of Brabant (Tilburg, the Netherlands) declared that the Medical Research Involving Human Subjects Act (WMO) did not apply to these studies (study numbers NW2024−44, NW2023−10). All procedures including informed consent were approved by our local Research Board and Board of Directors.

### Baseline characteristics

The questionnaire started with asking gender and year of birth. The **general hospital population** was asked additional information; level of education, type of contact with the hospital (hospital admission or outpatient visit) and a question about if anything has changed in their situation that may have influenced completion of the second questionnaire (No, nothing has changed, Yes, I am doing better/worse now). Educational level was categorized into high, middle and low level corresponding to the categorization used by the Dutch Central Bureau of Statistics (see Table 1). Participants were asked to report their highest completed level of education.

**Table 1:** Patient characteristics of the general hospital and lifestyle outpatient population.

	General hospital population			Lifestyle outpatient population		
		TEST	RETEST		TEST	RETEST
		n = 360	n = 247		n = 139	n = 121
		n(%)	n(%)		n(%)	n(%)
Gender (F)		178(49.4)	110(44.5)		89 (64.5)	75 (62.5)
Age (y)	mean ± SD	62.2 ± 13.0	64.1 ± 11.4	mean ± SD	52.89 ± 12.8	53.05 ± 13.3
	≤40	28(7.8)	12(4.9)	≤34	17(12.4)	15(12.5)
	41-60	103(28.6)	64(25.9)	35-64	92(67.2)	78(65.0)
	61-80	221(61.4)	164(66.4)	≥65	28(20.4)	27(22.5)
	≥ 81	8(2.2)	7(2.8)			
Education ^1^	Low	120 (33.3)	81 (32.8)		–	–
	Middle	105(29.2)	71(28.7)		–	–
	High	135(37.5)	95(38.5)		–	–
Hospital visit	Inpatient	50 (13.9)	30 (12.1)		–	–
	Outpatient	310 (86.1)	217 (87.9)		139 (100)	121 (100)

^1^Educational level. Low: primary education, prevocational secondary education, first three years of senior general secondary education, first three years of pre-university education and senior secondary vocational education level 1. Middle: four or five years of senior general secondary education, four, five or six years of pre university education and senior secondary vocational education, level 2, 3 or 4. High: higher vocational education and university education

### 22-item Positive Health questionnaire

The PH22 consists of 22 items, each scored on a 0–10 scale; “completely disagree” to “completely agree”, resulting in a total score ranging from 0 to 220 [11]. The PH22 comprises four dimensions: Physical Fitness (5 items; score range 0–50), Satisfaction with self, others and life (9 items; score range 0–90), Daily life management (5 items; score range 0–50), and Future perspective (3 items; score range 0–30) (see S1 Appendix S2). Simple-summated scoring (i.e., item responses within each scale are summed) is used to calculate dimension scores and total scores. The PH22 dimension and total scores are treated as continuous variables. Structural validity and internal consistency of the PH22, assessed using factor loadings, inter-item correlations, Cronbach’s α, and model fit indices, was found to be good in a general (Dutch) population [11].

### Statistical analyses

**Fig 1:**
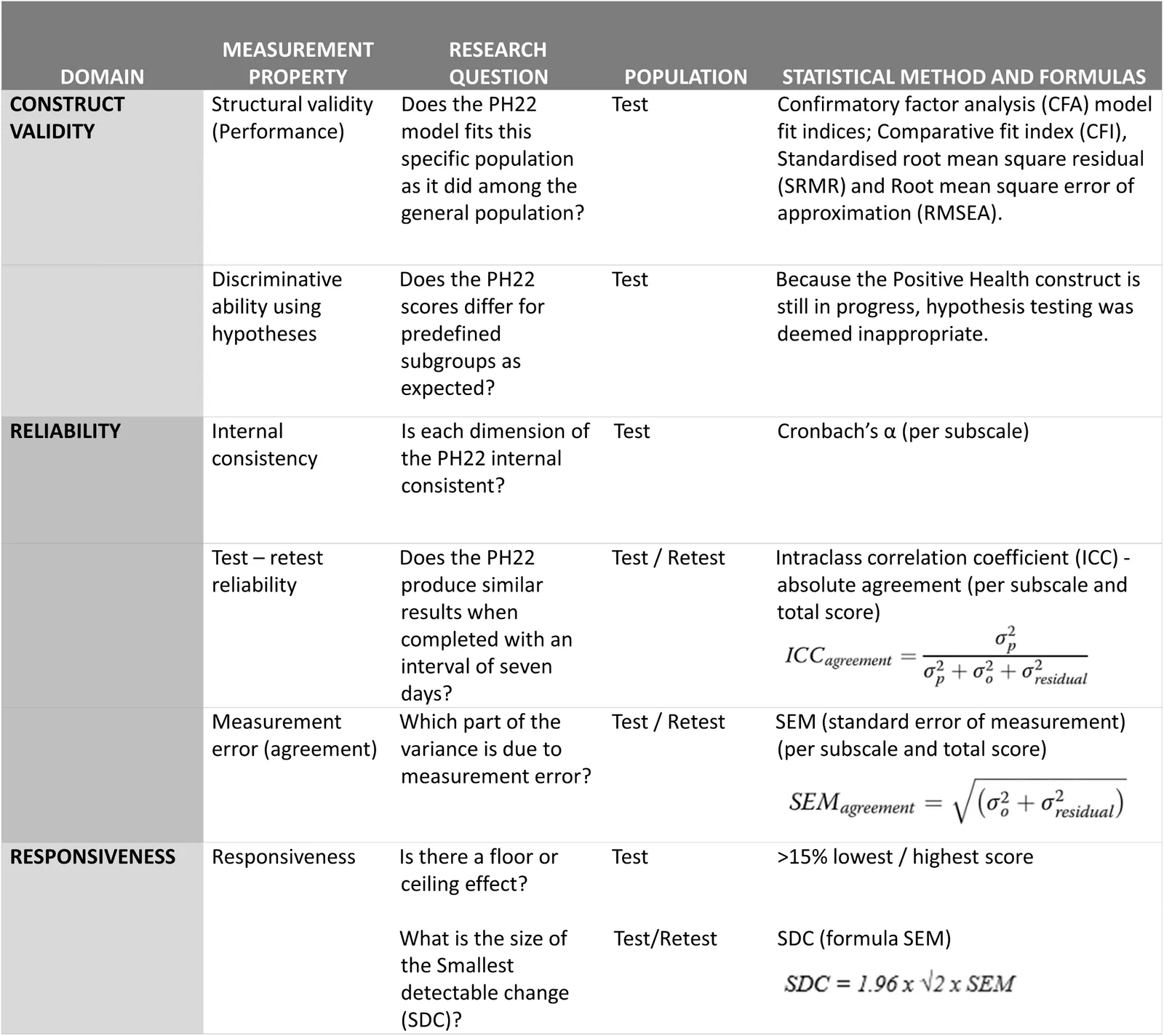
Research questions and accompanying measurement properties with statistical methods.

To analyze, in line with international guidelines [13–15], the reliability of the PH22, its performance and discriminative ability, measurement properties were explored for both the **general hospital population** and **Lifestyle outpatient population**, separately. For the general hospital population those implying anything had changed in their situation that may had influenced completion of the retest questionnaire were excluded for the test-retest reliability analyses. This was not applied among the other population because this information was not known. The specific measurement property with statistical method and formula per research question are presented in Fig 1. Confirmatory factor analysis (CFA) was conducted using the Lavaan package 0.6.14 in R [16]. All other statistical analyses were performed in SPSS v27.0.

### Structural validity (performance) and internal consistency

Prior to measurement property analyses, distribution of the item scores were assessed by inspection of the mean, median, their deviation measures, histogram and QQ-plots. Data were considered normally distributed if the mean and median were similar, histograms follow a symmetrical, bell-shaped form and QQ plots did not indicate systematic departures from normality [17]. Confirmatory factor analysis (CFA) with Maximum Likelihood estimation for continuous (non)normally distributed data was conducted [16] to assess if factor structures of the PH22 had an acceptable fit in these research populations [14]. Fit-parameters used were the chi-square (χ²), Comparative fit index (CFI), Standardised root mean square residual (SRMR) and Root mean square error of approximation (RMSEA). For CFI, values between 0.90–0.95 represent acceptable and >0.95 superior model fit [18,19]. For SRMR values ≤0.08 was considered good fit. For RMSEA values good fit was ≤ 0.05, between 0.05–0.08 acceptable fit, > 0.08 medium fit, and >0.1 poor fit [18,19]. Internal consistency was investigated using Cronbach’s α. Values between 0.7–0.9 were considered good and >0.9 as too high implying a redundancy of items [14].

### Test-retest reliability, measurement error and smallest detectable change (SDC)

Test-retest reliability was explored to assess if the PH22 produces similar results when repeated in identical circumstances. Used was the intraclass correlation coefficient (ICC) on absolute agreement, which includes error variance and systematic differences, to determine the level of agreement between measurements [14]. The ICC was calculated using variance components derived through the VARCOMP procedure in SPSS. This procedure provides estimates of the variance between patients (σ^2^ p), the variance due to systematic differences between observations (σ^2^ o), and the residual variance (σ^2^ residual) based on linear mixed modelling with restricted maximum likelihood estimation (see Formula Fig 1) [14]. Group level measurement ICC values between 0.7–0.9 represent good agreement and ≥0.9 perfect agreement; for individual level measurement (n = 1) an ICC value ≥0.9 is considered acceptable.

To assess how outcomes of repeated measurements completed in identical circumstances differ, the standard error of measurement (SEM) on agreement was used. The SEM-agreement includes error variance and systematic differences to determine the level of measurement error between measurements. SEM-agreement was calculated using the variance due to systematic differences between observations (σ^2^ o) and the residual variance (σ^2^ residual) (for formula see Fig 1) [14]. For interpretation, the SEM was also calculated as percentage of the possible score range of each dimension and total score. A percentage <10% was considered acceptable [20,21].

The smallest detectable change (SDC) was assessed to enhance interpretation of change scores. The SDC is defined as “the change beyond measurement error and outside the limits of agreement” [22]. The SDC was calculated using the SEM (see Fig 1). No threshold values for the SDC have been established in the literature. Because the SDC is derived from the SEM which in turn is related to the ICC, the SDC can be considered acceptable if the ICC and SEM are acceptable.

### Discriminative ability

Between-group differences were explored for educational level (low, middle, high) and by comparing the dimension and PH total scores of the two study populations with PH22 scores from a representative general Dutch population [11] categorized into no healthcare utilisation, one or more visits to the general practitioner, or one or more consultations with a healthcare specialist, during the past 12 months. PH22 scores for these subgroups were calculated by the first author (LNvV) and not published prior. Based on research in this field [10,23–25] it is to be expected that those higher educated, those not being (chronically) ill or making less use of healthcare facilities score higher for their PH. Therefore, it was hypothesised that the PH22 scores are higher for the higher educated subgroups and lower for the general hospital population and Lifestyle outpatient population compared to a Dutch population not visiting a general practitioner or healthcare specialist**.** Descriptive statistics (mean, median, standard deviation, minimum, maximum, percentiles (15, 85)) were reported per subgroup and visually compared to each other. Because the Positive Health construct is still in progress, hypothesis testing was deemed inappropriate.

### Study size

For a test-retest study, and correlations and comparisons between subgroups, a sample size of 50–100 (per subgroup) is recommended [13,14]. However, larger samples are preferred. To explore factor analyses 4–10 respondents per item of the questionnaire is recommended, with a minimum sample size of 100 [14,15]. These sample size seemed feasible. For the **general hospital population** a response rate of 10–30% was expected (based on earlier experiences in this hospital with comparable enrollment method); n = 200–600 (out of 2000). For the **Lifestyle outpatient population** the expected response rate was 60%; n = 120 (out of 200).

## Results

### Respondents

Among the **general hospital population** a total 2245 patients were informed about the study. Out of this group 567 patients (25.3%) were interested to participate and received more detailed information. Informed consent was given by 376 patients (response rate 66.3%), of which 16 persons did not answer (all) the items of the PH22, and were excluded from analyses. The final study population consisted of n = 360. The retest was performed by n = 323 (89.7%), of which n = 247 reported that nothing changed during the study period in relation to their Positive Health. Patient characteristics are shown in Table 1. No relevant differences were seen between the test and retest population.

Among the population visiting the **Lifestyle outpatient clinic** 159 patients were interested in participating and gave informed consent. 139 patients completed the PH22. 121 respondents completed the PH22 at the retest also. No differences were seen between the test and retest population (Table 1). For ten respondents the PH22 was not fully completed for the retest. Only data of completed dimensions were used. No data were imputed.

### Distribution of the PH22 scores

For the **general hospital population** (n = 360) scores were interpreted as nonnormally distributed (see S2 Appendix S3A for histogram and QQ plots, and S1 Appendix S6 for descriptive statistics,). The (sum)scores were skewed to the left and highly peaked: more outliers for the lower scores and higher frequency of scores around the mean, especially for the dimensions Contentment with self, others and life and Daily life management. No floor or ceiling effects (>15% obtains the or lowest or highest possible score, respectively) were present. Also, for the **lifestyle outpatient population** (n = 139) scores were interpreted as nonnormally distributed. Nonnormality was primarily based on the Histogram and QQ plots (see S3 Appendix S3B; especially for the dimension Daily life management and the total score. No floor or ceiling effects were present (S1 Appendix S6).

### Internal consistency

The fit indices of the four-factor structure of the PH22 among the **general hospital population** were adequate and ranged from; acceptable to good fit for both first and second order CFA (see S1 Appendix S4). The fit indices of the four-factor structure of the PH22 among the **lifestyle outpatient population** were less adequate and ranged from; (not) acceptable to no good fit for both first and second order CFA (see S1 Appendix S4). Among both populations Cronbach’s alpha’s were good for the dimensions Physical fitness, Contentment with self, others and life and Daily life management, but low (<0.7) for the dimension Future perspective (see Table 2 A and B).

**Table 2:** A. Reliability test characteristics of the PH22 of the general hospital population.

	Consistency	Test-Retest					
	n=360	n=247					
	CA	ICC	Mean difference ±SD	SEM	SEM%	SDC	SDC%
Positive Health (0-220)	-	0.873	-1.82 ±10.60	7.59	3.4	21.04	9.6
Physical fitness (0-50)	0.715	0.820	-0.98 ±3.77	2.75	5.5	7.62	15.2
Contentment (0-90)	0.916	0.858	-0.35 ±5.33	3.77	4.2	10.44	11.6
Self-management (0-50)	0.778	0.812	0.05 ±3.28	2.31	4.6	6.41	12.8
Future perspective (0-30)	0.684	0.634	-0.54 ±3.95	2.81	7.3	7.80	26.0
**B. Reliability test characteristics of the PH22 of the lifestyle outpatient population.**							
	Consistency	Test-Hertest					
	n=139	n=121					
	CA	ICC	Mean difference ±SD	SEM	SEM%	SDC	SDC%
Positive Health (0-220)	-	0.903	-4.28 ±10.90	8.26	3.8	22.89	10.4
Physical fitness (0-50)	0.721	0.842	1.17±4.09	3.09	6.2	8.57	17.1
Contentment (0-90)	0.877	0.880	-1.87 ±5.77	4.25	4.7	11.77	13.1
Self-management (0-50)	0.723	0.825	-0.67 ±4.35	3.09	6.2	8.58	17.2
Future perspective (0-30)	0.649	0.758	-0.90 ±3.12	2.29	7.6	6.36	21.2

CA; Cronbach’s alpha, ICC; Intraclass correlation coefficient, SD; standard deviation, SEM; standard error of measurement, SDC; smallest detectable change

### Test-retest reliability

For both populations ICCs were well above 0.7 indicating good agreement between test and retest. ICC was highest for the PH total score reaching perfect agreement (>0.9) among the **outpatient lifestyle population**. Exception was the dimension Future perspective (see Table 2 A-B). Among the **Lifestyle outpatient population** the ICC was still acceptable (0.75), but among the **general hospital population** the ICC was to low (<0.7), meaning large random variability. This was also reflected by the SEM which was largest among the dimension Future perspective, although still less than 10%.

### Discriminative ability

**Fig 2:**
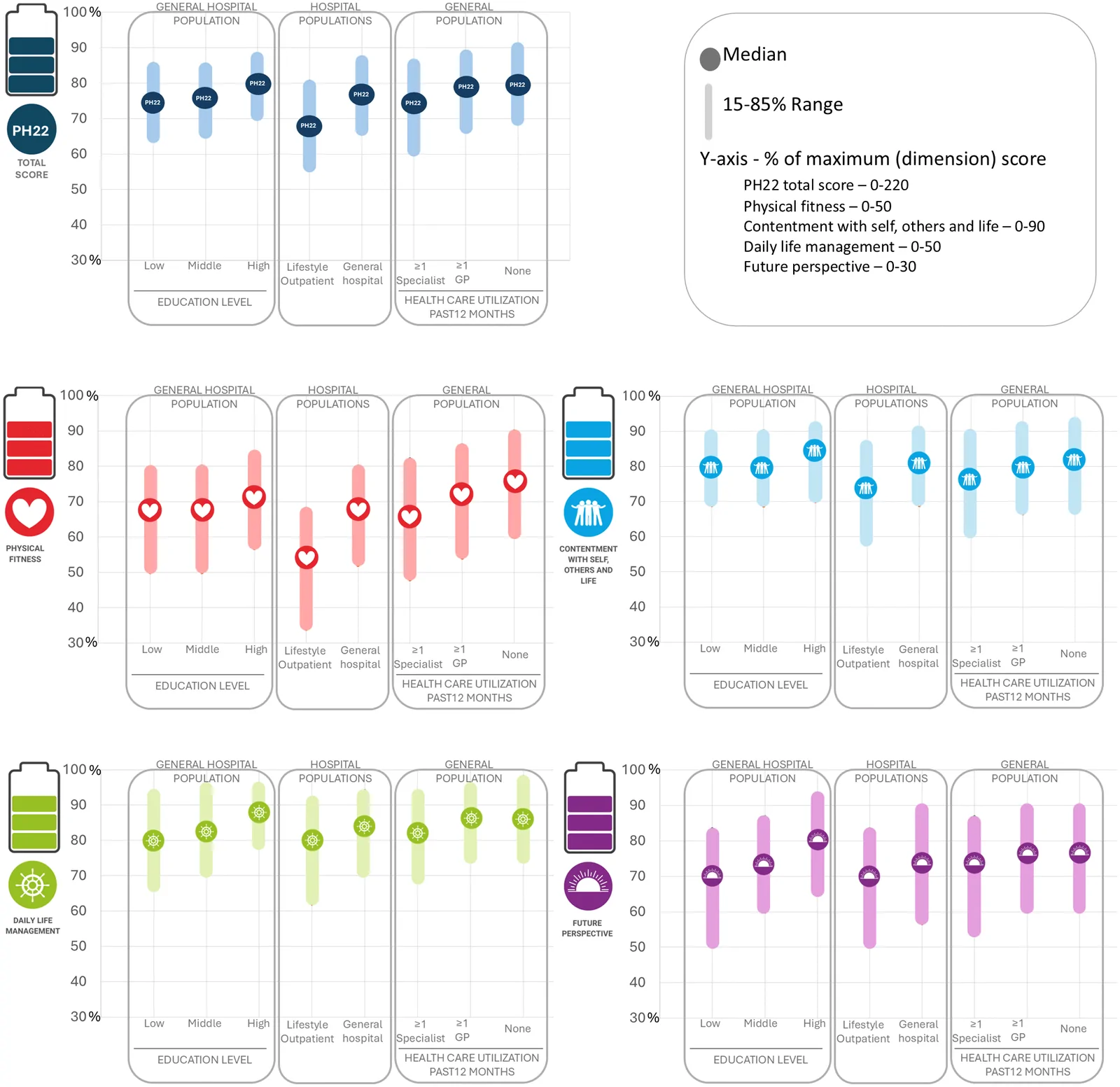
PH22 dimension and total scores in different settings and subgroups.

The expectations were confirmed. PH22 total and dimension scores were higher for the higher level of education subgroup (see Fig 2 and S1 Appendix S5 for numbers). For settings with more (chronic) disease or more healthcare use PH22 scores were consistently lower (Fig 2 and S1 Appendix S6).

## Discussion

This study showed good reliability, responsiveness, and sufficient performance for the PH22 among a general and more specific hospital population.

Two psychometric test results of the PH22 were moderate and less accurate than were seen among the general (Dutch) population [11]. This concerned its performance, in particular among the Lifestyle outpatient population, and internal consistency of the dimension ‘Future perspective’. In contrast to the adequate model fit of the PH22 model, according to the CFI, RMSEA and SRMR fit indices, among the general Dutch population [11] and general hospital population in this study, only the RMSEA showed an adequate model fit for the PH22 among the Lifestyle outpatient population. This might indicate that the four dimensions do not exactly assess the same construct for this specific population compared to a more general population. Internal consistency (Cronbach’s alpha) was < 0.7 for the dimension Future perspective indicating too low coherence among the items of this factor in this population. It can be hypothesized that patients, in particular patients with noncommunicable chronic diseases such as obesity, might be more short-term focused instead of future oriented due to other priorities or limited resources compared to healthier populations. This phenomenon was previously also seen among individuals with fewer resources or lower levels of education [26,27]. Although this difference might exist, the less accurate model fit and internal consistency were judged acceptable, since the deviations from the cut-off points were small with still adequate RMSEA. Moreover, the aim of the PH22 is to measure Positive Health scores at group level, and not for individual assessment such as diagnostics, in which exact model fit is less important [28]. However, caution should be taken comparing the PH22 scores between more specific groups. It should be noted that also other such as cultural differences can determine perceptions of health [29]. Cross-cultural validation is advisable.

Overall, measurement properties of the PH22 indicate good test-retest reliability (Intraclass correlation >0.7), a prerequisite to assess differences between and change within groups. Among the general hospital population, the ICC was too low for the dimension Future perspective, indicating large variability between measurements, reflected by a larger SEM, although still <10% from the total possible score. It should be realized that in case of a low ICC, sample sizes have to be very large for between group differences to be detected. ICC was highest for the total PH22 score reaching perfect agreement (>0.9) among the Lifestyle outpatient population, suggesting that the total PH22 score might also be applicable for individual level measurement (n = 1). However, since this finding was not consistent and model fit was moderate the PH22 is not recommended to use for individual level measurement. Also, the PH22 is not recommended as dialogue tool during consultation, the My Positive Heath (MPH) dialogue tool was developed for that aim [3].

This study shows that the reliability of the PH22 is good; in addition to validity, an important domain of measurement properties (COSMIN). Structural validity (i.e., performance) was already confirmed to be satisfactory among a general (Dutch) population [11], and is now shown to be also valid for general hospital populations. In developing the PH22, a rigorous combination of statistical item‑reduction procedures and expert‑guided content evaluation was used to overcome known limitations of earlier Positive Health instruments [11]. This process resulted in a concise and balanced set of items with broader content coverage and fewer redundancies, reflecting four meaningful dimensions aligned with the dynamic concept of Positive Health. In addition, this study confirmed satisfactory; test-retest reliability, responsiveness for change and discriminative ability. Also, the results of this study further strengthened its construct validity; as expected beforehand the PH22 scores were higher for patients with higher level of education and lower for those being (chronically) ill. To assign qualitative meaning to the differences in PH22 scores, it is of major interest for future research to define various facets of clinical relevance [30] and model clinical and commonly understood self-reported outcomes and align these. This will further improve the interpretability of the PH22 scores and refine the construct Positive Health.

Other recently published questionnaires to measure broad health concepts such as the Context-sensitive Positive Health Questionnaire (CPHQ2.0) [31–33] and I.ROC12 [9,10] focus on comparable but also other constructs than Positive Health; the capability approach [34] and personal recovery [35], respectively. It is of interest to explore which instrument serves which aim and context best. Future choices of which tool to use should not only depend on the measurement properties and usability of each tool but also on which construct definition is preferred as the outcome to measure. The PH22 is a suitable instrument for measuring Positive Health in scientific and policy-oriented evaluations of the Positive Health approach or patient-centered care.

### Strengths and limitations

This is the first study that explored reliability and performance of the PH22 among hospital populations. Some limitations were faced. To assess test-retest reliability it is recommended to exclude those who implied their situation had changed. For the Outpatient lifestyle population this information was unknown. Most likely, this would have resulted in less variability and better reliability properties. Another limitation might have been the smaller sample size of the Outpatient lifestyle population; individual cutoffs like for fit indices depend on the sample size and seem to perform reasonably well only with moderate sample sizes (i.e., n = 300; 700)[23]. This might (partly) influence the model fit seen among the Outpatient lifestyle population.

## Conclusion

This study is an addition to the development and validation study of the PH22. The found measurement properties indicate that the PH22 is usable to explore within and between group differences in Positive Health scores in scientific and policy-oriented evaluations of Positive Health and patient-centered approaches among healthcare settings. Caution should be taken among more specific (patients) groups, especially for the dimension Future perspective. Overall, the PH22 appears to be a reliable and applicable instrument for measuring Positive Health. Future research should focus on the clinical relevance and interpretation of different PH22 scores.

## Supporting information

S1 Appendix S1. Referral criteria for the Lifestyle outpatient clinic. S2. The 22 item self-reported Positive Health questionnaire (PH22). S4. Fit indices for the four-factor structure of the PH22 model. S5. PH22 scores in subgroups with different level of education among the general hospital population. S6. PH22 scores among different settings representing level of (chronic) disease or healthcare utilization.
(PDF)

S2 Appendix S3A. Histograms and QQ Plots of the PH22 scores of the general hospital population (n = 360). (PDF)

S3 Appendix S3B. Histograms and QQ Plots of the PH22 scores of the lifestyle outpatient population (n = 139). (PDF)

## References

[1] de Bekker A, Beijer M, Lemmens L. Towards an integrative approach of healthcare: implementing positive health in three cases in the Netherlands. BMC Health Serv Res. 2024;24(1):882. doi: 10.1186/s12913-024-11247-x 39095783 PMC11295315

[2] van Wietmarschen HA, Staps S, Meijer J, Flinterman JF, Jong MC. The use of the bolk model for positive health and living environment in the development of an integrated health promotion approach: a case study in a socioeconomically deprived neighborhood in The Netherlands. Int J Environ Res Public Health. 2022;19(4):2478. doi: 10.3390/ijerph19042478 35206663 PMC8879013

[3] van Vliet M, de Kleijn M, van den Brekel-Dijkstra K, Huijts T, van Hogen-Koster S, Jung HP, et al. Rapid review on the concept of positive health and its implementation in practice. Healthcare (Basel). 2024;12(6):671. doi: 10.3390/healthcare12060671 38540636 PMC10970180

[4] Huber M, Knottnerus JA, Green L, van der Horst H, Jadad AR, Kromhout D. How should we define health?. BMJ (Clinical research ed). 2011;343:d4163. 10.1136/bmj.d4163 21791490

[5] Huber M, van Vliet M, Giezenberg M, Winkens B, Heerkens Y, Dagnelie PC, et al. Towards a “patient-centred” operationalisation of the new dynamic concept of health: a mixed methods study. BMJ Open. 2016;6(1):e010091. doi: 10.1136/bmjopen-2015-010091 26758267 PMC4716212

[6] Lemmens L, de Bruin S, Beijer M, Hendrikx R, Baan C. Het gebruik van brede gezondheidsconcepten, inspirerend en uitdagend voor de praktijk. Bilthoven: Rijksinstituut voor Volksgezondheid en Milieu; 2019.

[7] Doornenbal BM, Vos RC, Van Vliet M, Kiefte-De Jong JC, van den Akker-van Marle ME. Measuring positive health: concurrent and factorial validity based on a representative Dutch sample. Health Soc Care Commun. 2022;30(5):e2109–17. doi: 10.1111/hsc.13649 34791738 PMC9544585

[8] Van Vliet M, Doornenbal BM, Boerema S, van den Akker-van Marle EM. Development and psychometric evaluation of a Positive Health measurement scale: a factor analysis study based on a Dutch population. BMJ Open. 2021;11(2):e040816. doi: 10.1136/bmjopen-2020-040816 33550237 PMC7925905

[9] van Druten VP, Metz MJ, Mathijssen JJP, van de Mheen D, van Vliet M, Rudd B, et al. Measuring Positive Health using the My Positive Health (MPH) and Individual Recovery Outcomes Counter (I.ROC) dialogue tools: a panel study on measurement properties in a representative general dutch population. Appl Res Quality Life. 2024;21:2825 –2846.doi: 10.1007/s11482-024-10356-3

[10] van Druten VP, Nahar-van Venrooij LMW, Tiemens BG, van de Mheen D, de Vries E, Metz MJ. Construct validity of the measurement tools PH42 and I.ROC12 to measure positive health in a general population. Health Care Anal. 2025. doi: 10.1007/s10728-025-00533-2 40742498 PMC13675334

[11] Nahar-van Venrooij LMW, Metz MJ, van Vliet M, van Druten VP, van der Zwaard BC. Development and cross-validation of a short questionnaire to evaluate self-reported positive health: a cross-sectional panel study of structural validity among a general Dutch population. BMJ Open. 2025;15(3):e091377. doi: 10.1136/bmjopen-2024-091377 40132829 PMC11934353

[12] Institute for Positive Health. Positieve gezondheid. Wat is het?. 2019. https://www.iph.nl/positieve-gezondheid/wat-is-het/

[13] Gagnier JJ, Lai J, Mokkink LB, Terwee CB. COSMIN reporting guideline for studies on measurement properties of patient-reported outcome measures. Qual Life Res. 2021;30(8):2197–218. doi: 10.1007/s11136-021-02822-4 33818733

[14] de Vet HCW, Terwee CB, Mokkink LB, Knol DL. Measurement in medicine - a practical guide. Cambridge University Press; 2011.

[15] Mokkink LB, de Vet HCW, Prinsen CAC, Patrick DL, Alonso J, Bouter LM, et al. COSMIN risk of bias checklist for systematic reviews of patient-reported outcome measures. Qual Life Res. 2018;27(5):1171–9. doi: 10.1007/s11136-017-1765-4 29260445 PMC5891552

[16] Rosseel Y. An R package for structural equation modeling. J Stat Softw. 2012;48(2).

[17] Petrie A, Sabin C. Medical statistics at a glance. 3rd ed. UK: Oxford; 2009.

[18] Byrne BM. Structural equation modeling with AMOS. 2nd ed. Psychology Press; 2013.

[19] Hu L, Bentler PM. Cutoff criteria for fit indexes in covariance structure analysis: Conventional criteria versus new alternatives. Struct Equat Model: A Multidiscip J. 1999;6(1):1–55. doi: 10.1080/10705519909540118

[20] Braun T, Ehrenbrusthoff K, Bahns C, Happe L, Kopkow C. Cross-cultural adaptation, internal consistency, test-retest reliability and feasibility of the German version of the evidence-based practice inventory. BMC Health Serv Res. 2019;19(1):455. doi: 10.1186/s12913-019-4273-0 31277662 PMC6612094

[21] van Baalen B, Odding E, van Woensel MPC, Roebroeck ME. Reliability and sensitivity to change of measurement instruments used in a traumatic brain injury population. Clin Rehabil. 2006;20(8):686–700. doi: 10.1191/0269215506cre982oa 16944826

[22] de Vet HCW, Terwee CB, Mokkink LB, Knol DL. Measurement in medicine - a practical guide. Cambridge University Press; 2011.

[23] IHME-CHAIN Collaborators. Effects of education on adult mortality: a global systematic review and meta-analysis. Lancet Public Health. 2024;9(3):e155–65. doi: 10.1016/S2468-2667(23)00306-7 38278172 PMC10901745

[24] Wang T, Li Y, Zheng X. Association of socioeconomic status with cardiovascular disease and cardiovascular risk factors: a systematic review and meta-analysis. Z Gesundh Wiss. 2023;:1–15. doi: 10.1007/s10389-023-01825-4 36714072 PMC9867543

[25] Mielck A, Reitmeir P, Vogelmann M, Leidl R. Impact of educational level on health-related quality of life (HRQL): results from Germany based on the EuroQol 5D (EQ-5D). Eur J Public Health. 2013;23(1):45–9. doi: 10.1093/eurpub/ckr206 22434205

[26] Guthrie LC, Butler SC, Ward MM. Time perspective and socioeconomic status: a link to socioeconomic disparities in health?. Social Sci Med. 2009;68(12). 10.1016/j.socscimed.2009.04.004 PMC2711848 19394738

[27] Shu L, Gong T, Wang Y, Li Q, Xie Z. The negative association of low subjective socioeconomic status with future orientation: the protective role of low fatalism. BMC Psychol. 2024;12(1):664. doi: 10.1186/s40359-024-02173-y 39548581 PMC11568625

[28] Goretzko D, Siemund K, Sterner P. Evaluating model fit of measurement models in confirmatory factor analysis. Educ Psychol Meas. 2024;84(1):123–44. doi: 10.1177/00131644231163813 38250508 PMC10795573

[29] van Druten VP, Bartels EA, van de Mheen D, de Vries E, Kerckhoffs APM, Nahar-van Venrooij LMW. Concepts of health in different contexts: a scoping review. BMC Health Serv Res. 2022;22(1):389. doi: 10.1186/s12913-022-07702-2 35331223 PMC8953139

[30] Rathgeber IS, Krepper D, Wintner LM, Giesinger JM, Sztankay M. Unraveling assumptions about clinical relevance in patient-reported outcome data : a qualitative study on perspectives of different interest groups. Support Care Cancer. 2026;34(2):155. doi: 10.1007/s00520-026-10387-6 41627518 PMC12864281

[31] Boelens M, Roumen C, van Gurp EJB, Dierx J, Nahar - van Venrooij L MW, van Zutphen T, et al. Stakeholder and end-user perspectives to improve the context-sensitive positive health questionnaire (CPHQ) to measure broad health. medRxiv. 2025. 10.1371/journal.pone.0330014 PMC13502646 42636186

[32] Doornenbal BM, van Zutphen T, Beumeler LFE, Vos RC, Derks M, Haisma H, et al. Development and validation of a Context-sensitive Positive Health Questionnaire (CPHQ): a factor analysis and multivariate regression study. J Patient Rep Outcomes. 2024;8(1):44. doi: 10.1186/s41687-024-00718-8 38607610 PMC11014831

[33] Dubbeldeman EM, Boelens M, Gurp EJB-v, Dierx J, Spreeuwenberg MD, Jong JCK-d. A longitudinal psychometric evaluation of a context-sensitive positive health questionnaire for measuring broad health in Dutch adults. MedRxiv. 2025. 10.1186/s41687-026-01098-x PMC13454085 42230519

[34] Chiappero-Martinetti E, Venkatapuram S. The capability approach: a framework for population studies. APS. 2014;28(2):708. doi: 10.11564/28-2-604

[35] Monger B, Hardie SM, Ion R, Cumming J, Henderson N. The individual recovery outcomes counter: preliminary validation of a personal recovery measure. Psychiatrist. 2013;37(7):221–7. doi: 10.1192/pb.bp.112.041889

