# Supplementary material for: The 22-item Positive Health questionnaire (PH22) is reliable and applicable among different (Dutch) hospital populations: A test-retest study design
Source: PLoS One. 2026 Aug 20;21(8):e0356368. doi: 10.1371/journal.pone.0356368 (PMC13492778; doi:10.1371/journal.pone.0356368)
Supplement: Supplementary file 1 [file pone.0356368.s001.pdf]

S1. Referral criteria for the Lifestyle outpatient clinic

**Inclusion criteria**	
Patient BMI >25	✔
Lifestyle is a contributing factor to the patient's disease burden for which they are referred to the hospital	✔
The patient is able to independently participate in the consultation and the follow-up lifestyle improvement program	✔
**Exclusion criteria**	
Patient age / lifestyle improvement must provide added value. For patients over 70, assess what is still feasible in terms of physical activity and healthy nutrition/hydration	✖ ✔
The patient is not motivated: lifestyle improvement requires personal responsibility and effort from the patient	✖
The patient is already participating in or eligible for a cardiopulmonary or oncology rehabilitation program (participation after completion is of course possible)	✖
A medical procedure, surgery, or treatment is planned in the short term that would hinder a lifestyle intervention (e.g., limited mobility after a procedure)	✖

S2. The 22 item self-reported Positive Health questionnaire (PH22)

Item
Physical fitness
I feel fit
I have no physical complaints or pain
I sleep well
I eat well
I recover quickly after exercise, such as sports
Contentment with self, others and life
I accept myself for who I am
I feel in control of my life
In the mornings. I look forward to the day ahead
I accept life as it comes
I feel safe
I am content with where and with whom I live
I am in good contact with other people
I have people who support me when I need it
I consider my job or other activities to be meaningful
Daily life management
I am well capable of looking after myself. for example with regard to personal hygiene. getting dressed. shopping. cooking
I know my limitations
I am well capable of planning my day
I am well capable of managing the money that I have each month
I know how to apply for benefits or getting assistance from official agencies when necessary
Future perspective
I look for solutions to change difficult situations
I have ideals that I would like to achieve
I want to continue learning throughout my life

S4. Fit indices for the four-factor structure of the PH22 model

		X2, p	CFI[1]	RMSEA[2]	SRMR[3]
General hospital population (n=360)	Fit	-	Acceptable	Acceptable	Good
	First order CFA	p <=0.001	0.912	0.068 (0.058-0.078)	0.062
	Second order CFA	p<= 0.001	0.910	0.069 (0.059-0.079)	0.064
Lifestyle outpatient population (n=247)	Fit	-	Not acceptable	Acceptable	Not good
	First order CFA	p <= 0.001	0.868	0.074 (0.055-0.091)	0.089
	Second order CFA	p <= 0.001	0.867	0.074 (0.055-0.091)	0.900

[1] For [1]CFI, values between 0.90-0.95 represent acceptable and >0.95 superior model fit. For [2] RMSEA values good fit was ≤0.05, between 0.05-0.08 acceptable fit, >0.08 medium fit, and >0.1 poor fit. For [3] SRMR values ≤0.08 was considered good fit.

S5. PH22 scores in subgroups with different level of education among the general hospital population

Level of education			Total score PH22 (0-220)	Score Physical fitness (0-50)	Score Contentment with self, others and life (0-90)	Score Daily life management (0-50)	Score Future perspective (0-30)
Low[1]	n	120					
	Mean		162.55	32.63	70.25	39.60	20.07
	Median		164.50	34.00	72.00	40.00	21.00
	Std. Deviation		26.500	7.399	12.223	6.573	4.773
	Skewness		-1.238	-.885	-1.793	-.910	-.771
	Kurtosis		2.682	1.390	6.424	1.374	.626
	Minimum		51	5	6	15	4
	Maximum		204	49	90	50	29
	Percentiles	15	140.30	25.00	62.00	33.00	15.00
		85	188.00	39.85	81.00	47.00	25.00
Middle[2]	n	105					
	Mean		165.87	33.15	70.36	40.72	21.63
	Median		168.00	34.00	72.00	41.00	22.00
	Std. Deviation		24.620	7.059	12.041	6.443	4.414
	Skewness		-.890	-.330	-1.455	-.904	-.794
	Kurtosis		2.224	-.280	3.900	1.081	1.776
	Minimum		73	16	22	18	6
	Maximum		214	47	90	50	30
	Percentiles	15	142.90	24.90	61.90	35.00	18.00
		85	187.20	40.00	81.10	48.00	26.00
High[3]	n	135					
	Mean		175.09	34.86	73.79	42.96	23.48
	Median		177.00	35.00	76.00	44.00	24.00
	Std. Deviation		20.001	6.856	10.357	4.544	4.328
	Skewness		-.617	-.407	-1.441	-.772	-1.220
	Kurtosis		.425	.106	3.818	.355	2.626
	Minimum		113	15	24	28	5
	Maximum		220	50	90	50	30
	Percentiles	15	154.40	28.40	63.00	39.00	19.40
		85	194.00	42.00	83.00	48.00	28.00

Educational level. [1] Low: primary education, prevocational secondary education, first three years of senior general secondary education, first three years of pre-university education and senior secondary vocational education level 1. [2] Middle: four or five years of senior general secondary education, four, five or six years of pre university education and senior secondary vocational education, level 2, 3 or 4. [3] High: higher vocational education and university education

S6. PH22 scores among different settings representing level of (chronic) disease or healthcare utilization

Setting			Total score PH22 (0-220)	Score Physical fitness (0-50)	Score Contentment with self, others and life (0-90)	Score Daily life management (0-50)	Score Future perspective (0-30)
Lifestyle[1]	n	139					
	Mean		149.9	25.7	65.1	38.6	20.5
	Median		151.0	26.0	67.0	40.0	21.0
	Std. Deviation		27.04	7.93	13.48	7.60	4.75
	Skewness		-.552	-.193	-.730	-.714	-.550
	Kurtosis		.145	-.227	.932	.385	.839
	Minimum		66	4	17	15	5
	Maximum		215	47	90	50	30
	Percentiles	15	122.0	17.0	52.0	31.0	15.0
		85	177.0	34.0	78.0	46.0	25.0
Hospital[2]	n	360					
	Mean		168.22	33.62	71.61	41.19	21.80
	Median		171.00	34.00	73.00	42.00	22.00
	Std. Deviation		24.245	7.148	11.594	6.012	4.719
	Skewness		-1.070	-.568	-1.596	-1.031	-.863
	Kurtosis		2.503	.558	4.947	1.615	1.170
	Minimum		51	5	6	15	4
	Maximum		220	50	90	50	30
	Percentiles	15	145.15	26.00	62.00	35.00	17.00
		85	191.85	40.00	82.00	47.00	27.00
SHC[3]	n	977					
	Mean		161.31	32.40	67.31	40.47	21.13
	Median		165.00	33.00	69.00	41.00	22.00
	Std. Deviation		28.096	8.387	13.446	6.632	4.868
	Skewness		-.681	-.390	-.840	-.898	-.462
	Kurtosis		.592	.058	.932	.980	.049
	Minimum		59	3	9	14	5
	Maximum		220	50	90	50	30
	Percentiles	15	132.00	24.00	54.00	34.00	16.00
		85	189.00	41.00	81.00	47.00	26.00
GP[4]	n	705					
	Mean		170.74	35.51	70.91	42.15	22.17
	Median		174.00	36.00	72.00	43.00	23.00
	Std. Deviation		25.520	7.669	11.962	5.803	4.694
	Skewness		-.704	-.467	-.956	-.854	-.834
	Kurtosis		.713	.185	1.436	.680	.963
	Minimum		67	7	11	17	3
	Maximum		220	50	90	50	30

	Percentiles	15	145.90	27.00	59.90	37.00	18.00
		85	195.10	43.00	83.00	48.00	27.00
No[5]	n	751					
	Mean		174.87	37.73	72.24	42.43	22.46
	Median		177.00	38.00	74.00	43.00	23.00
	Std. Deviation		25.334	7.244	12.002	5.896	4.642
	Skewness		-.802	-.565	-.991	-1.053	-.838
	Kurtosis		.994	.358	1.326	1.593	.905
	Minimum		72	10	25	14	5
	Maximum		220	50	90	50	30
	Percentiles	15	150.80	30.00	60.00	37.00	18.00
		85	200.00	45.00	84.00	49.00	27.00

[1] Lifestyle outpatient population with BMI>25; [2]Hospital population 1 day after visit or after discharge; [3] Specialized health care, or [4]general practitioner (1 or more visits past 12 months) or [5] no health care use.
