# Supplementary material for: The 22-item Positive Health questionnaire (PH22) is reliable and applicable among different (Dutch) hospital populations: A test-retest study design
Source: PLoS One. 2026 Aug 20;21(8):e0356368. doi: 10.1371/journal.pone.0356368 (PMC13492778; doi:10.1371/journal.pone.0356368)
Supplement: Supplementary file 2 [file pone.0356368.s002.pdf]

Histogram

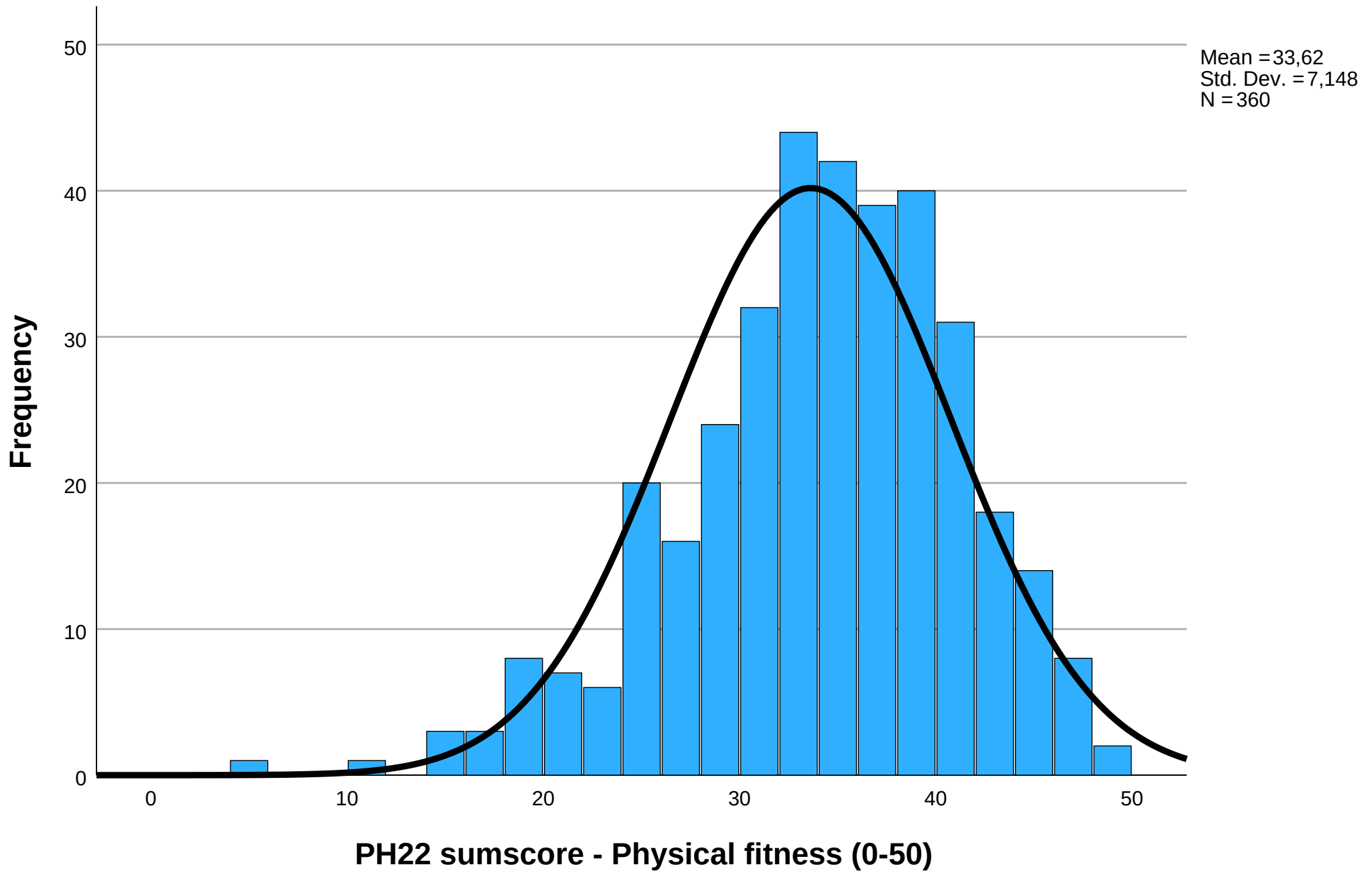
Mean =33,62
Std. Dev. =7,148
N =360
Frequency
0
10
20
30
40
50
PH22 sumscore - Physical fitness (0-50)

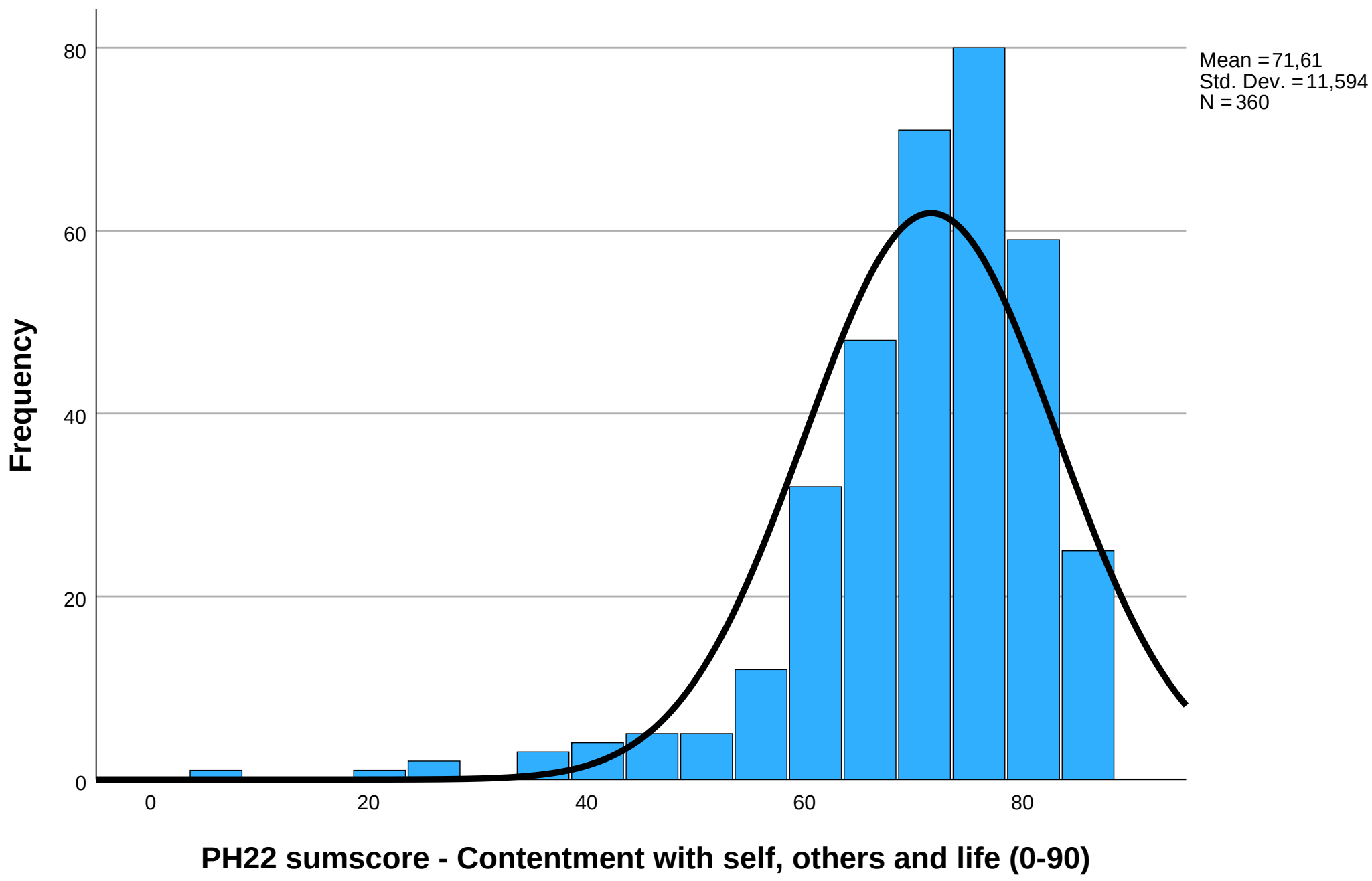
Mean =71,61
Std. Dev. =11,594
N =360
Frequency
0
20
40
60
80
PH22 sumscore - Contentment with self, others and life (0-90)

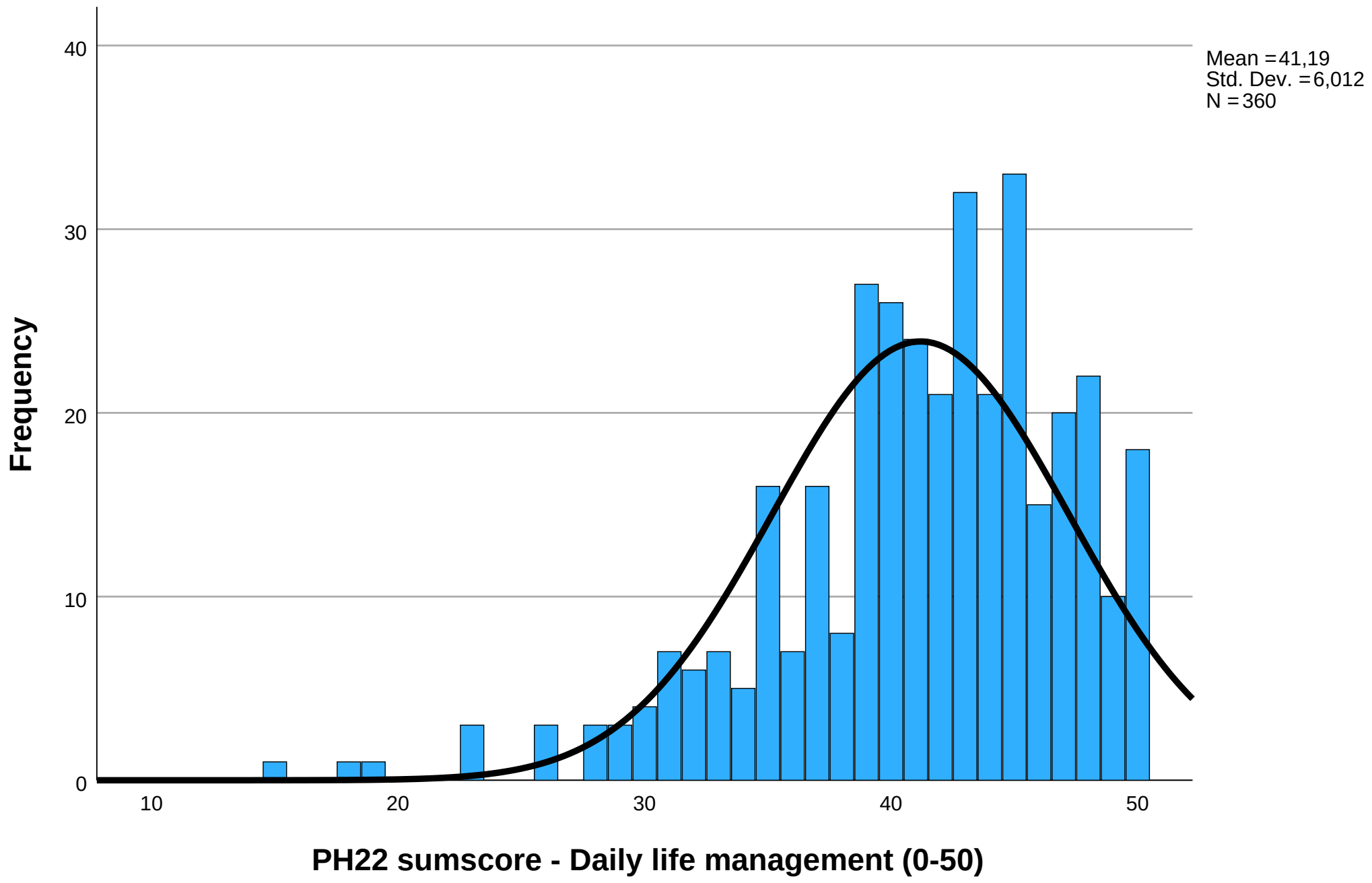
Mean =41,19
Std. Dev. =6,012
N =360
Frequency
40
30
20
10
0
10
20
30
40
50
PH22 sumscore - Daily life management (0-50)

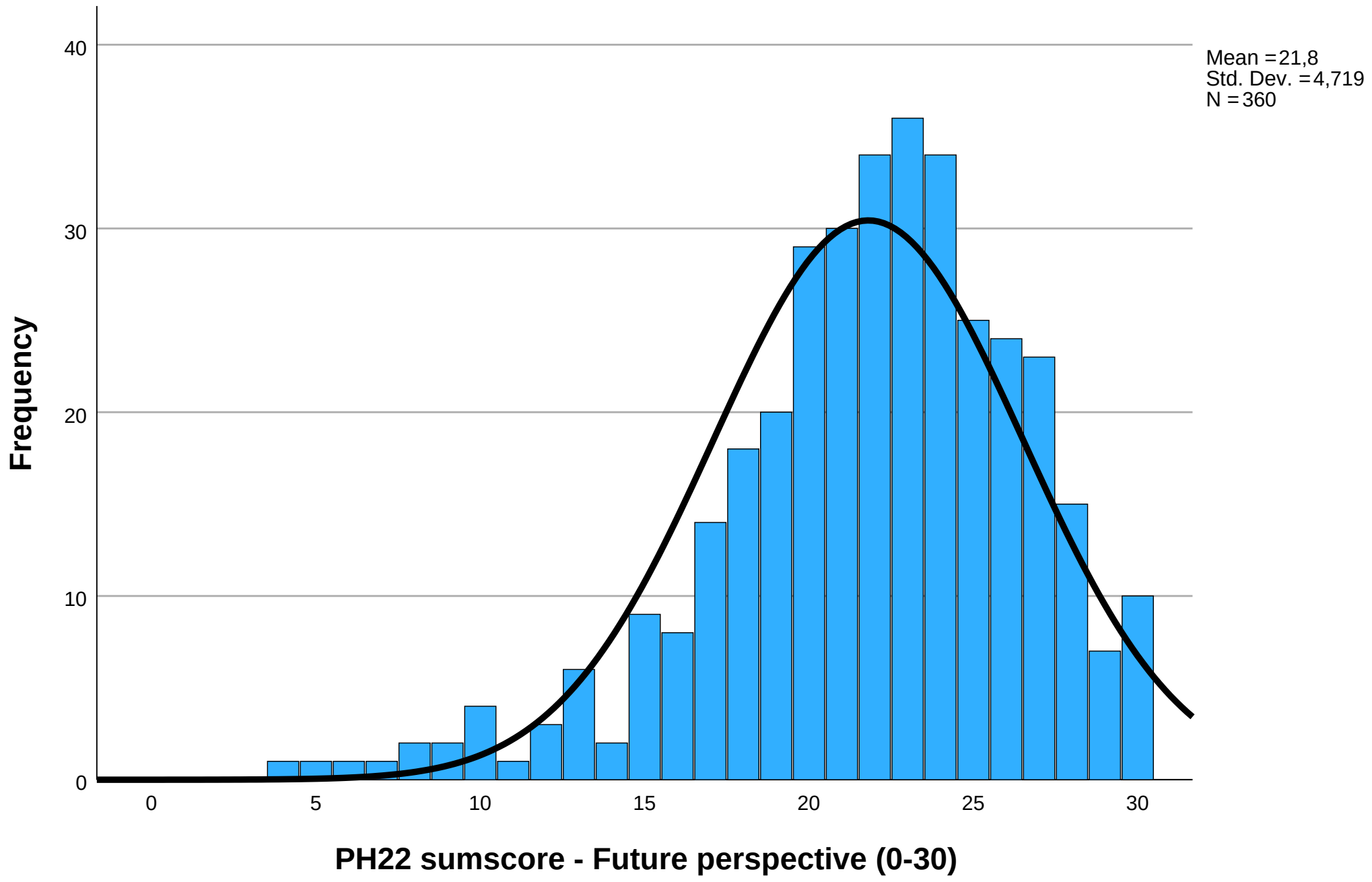
Mean =21,8
Std. Dev. =4,719
N =360
Frequency
40
30
20
10
0
0
5
10
15
20
25
30
PH22 sumscore - Future perspective (0-30)

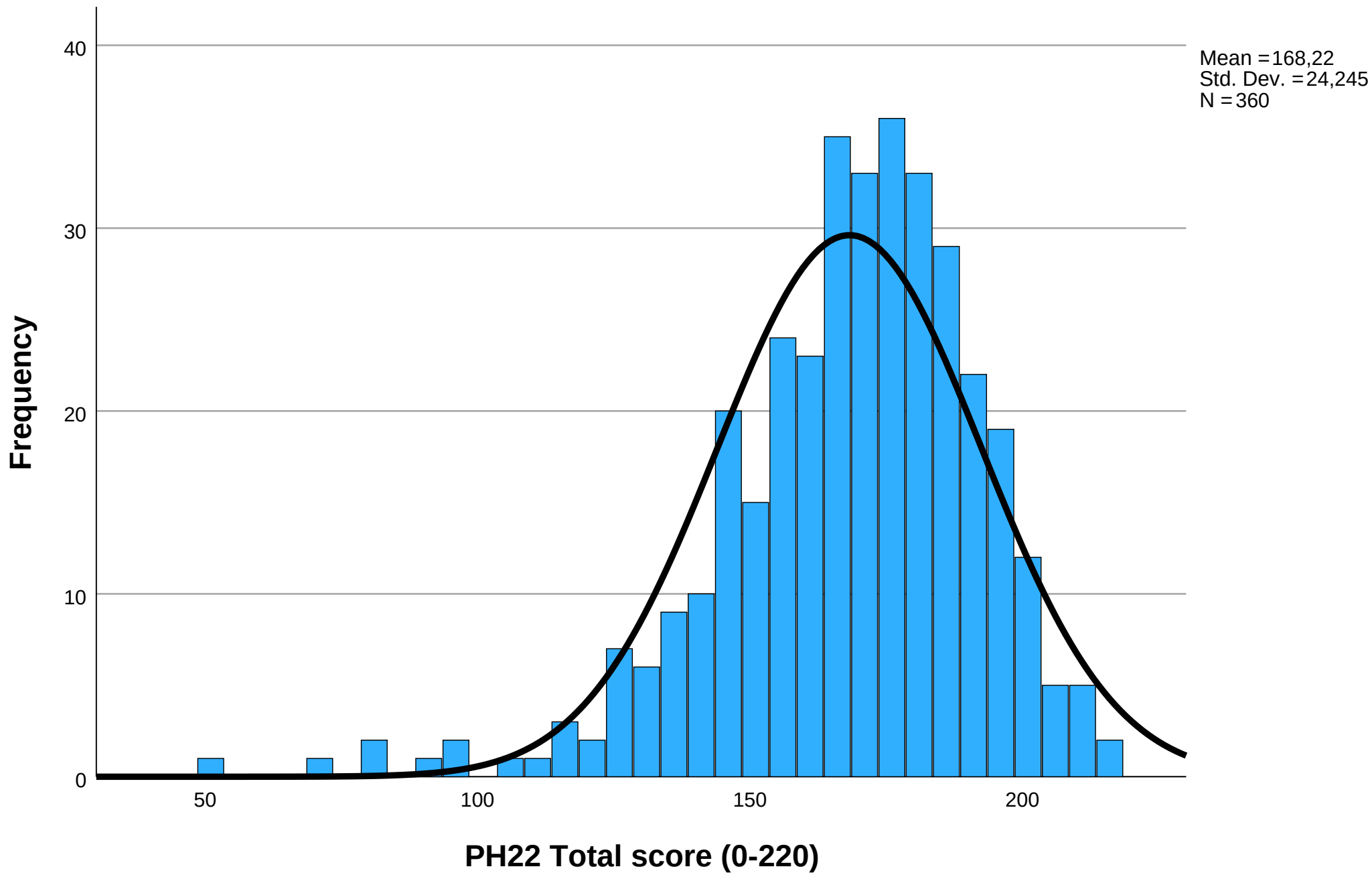

Normal Q-Q Plot

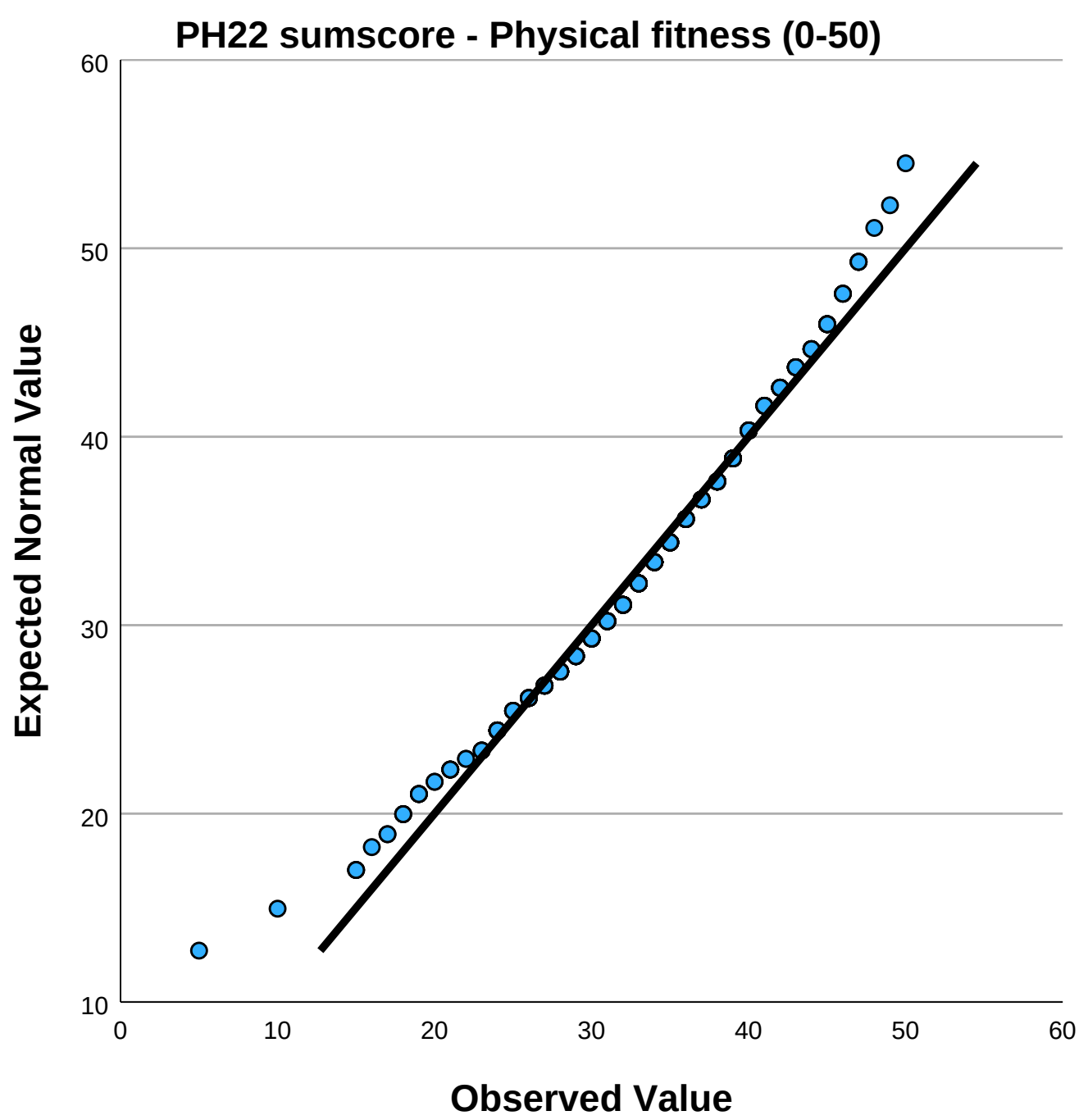

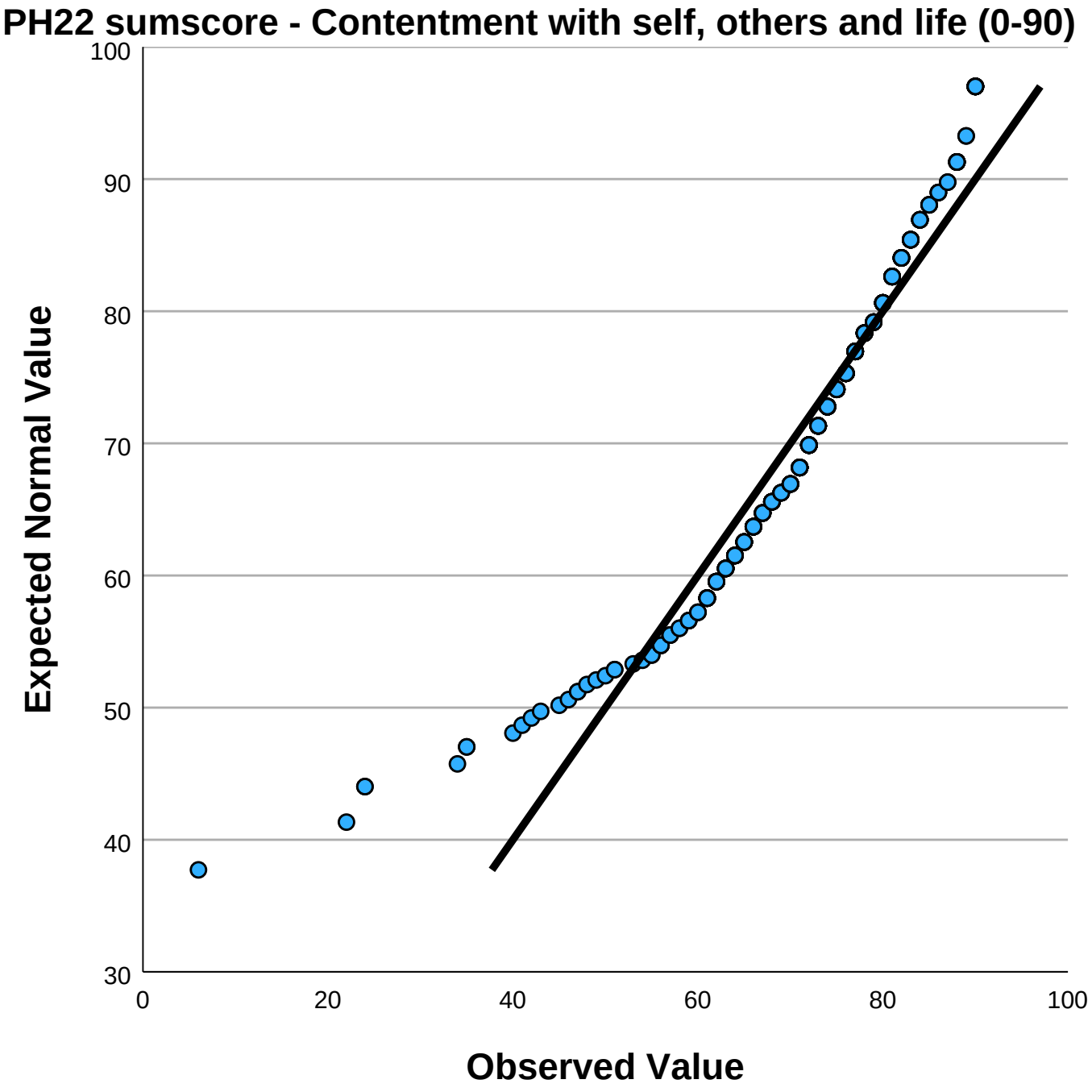
PH22 sumscore - Contentment with self, others and life (0-90)
Expected Normal Value
Observed Value
100
90
80
70
60
50
40
30
0
20
40
60
80
100

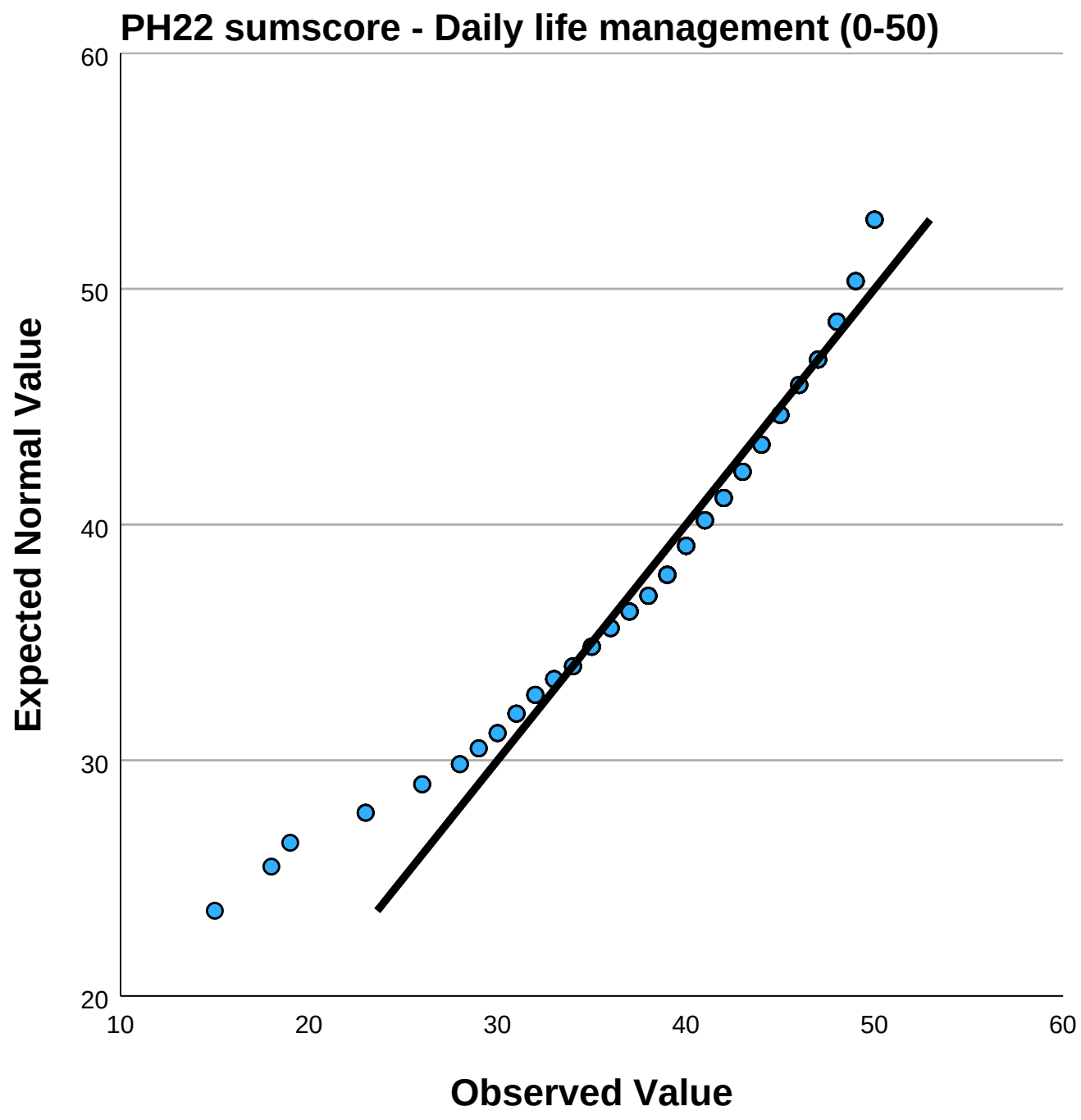
PH22 sumscore - Daily life management (0-50)
Expected Normal Value
Observed Value
60
50
40
30
20
10
20
30
40
50
60

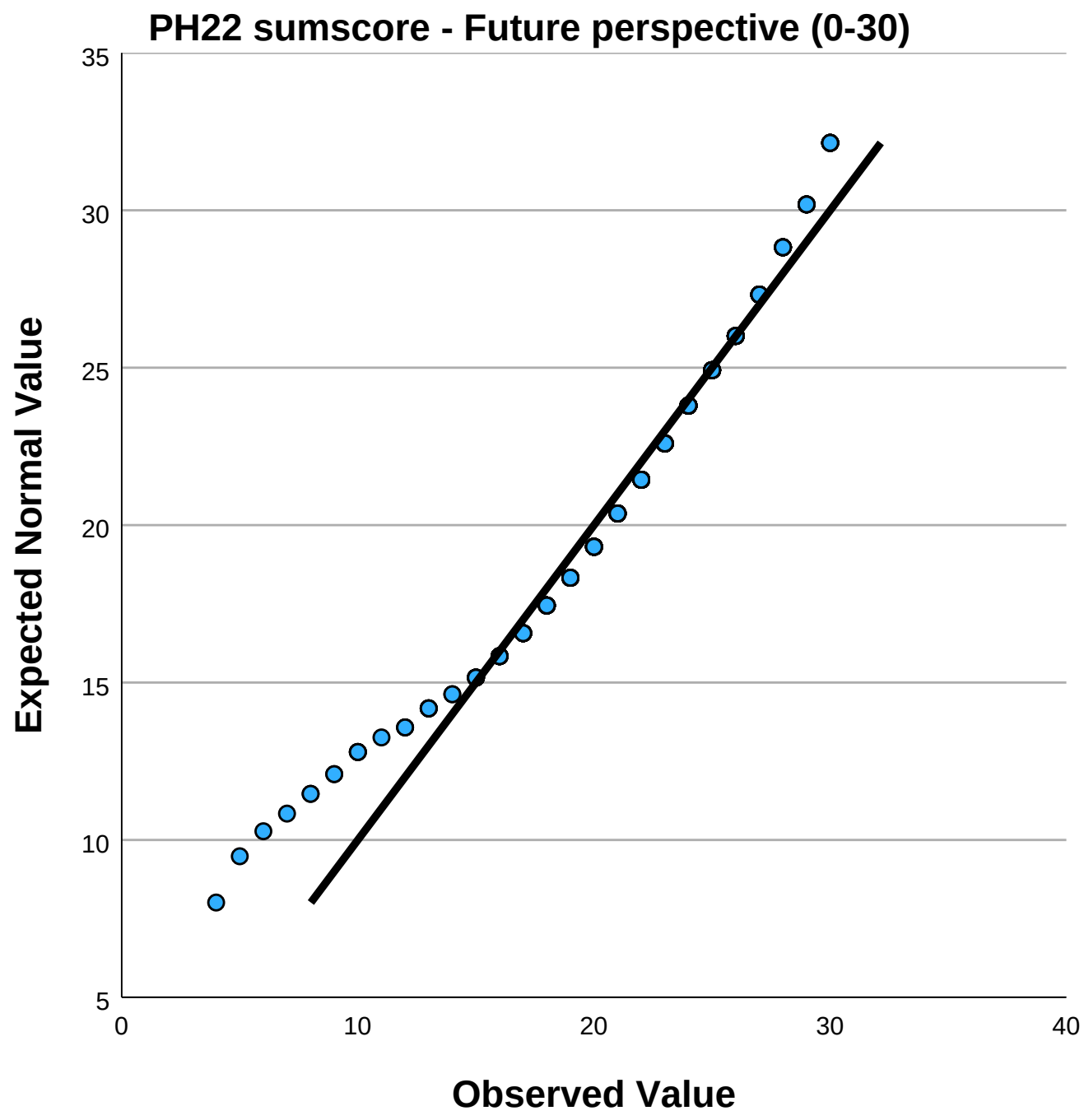
PH22 sumscore - Future perspective (0-30)
Expected Normal Value
Observed Value
35
30
25
20
15
10
5
0
10
20
30
40

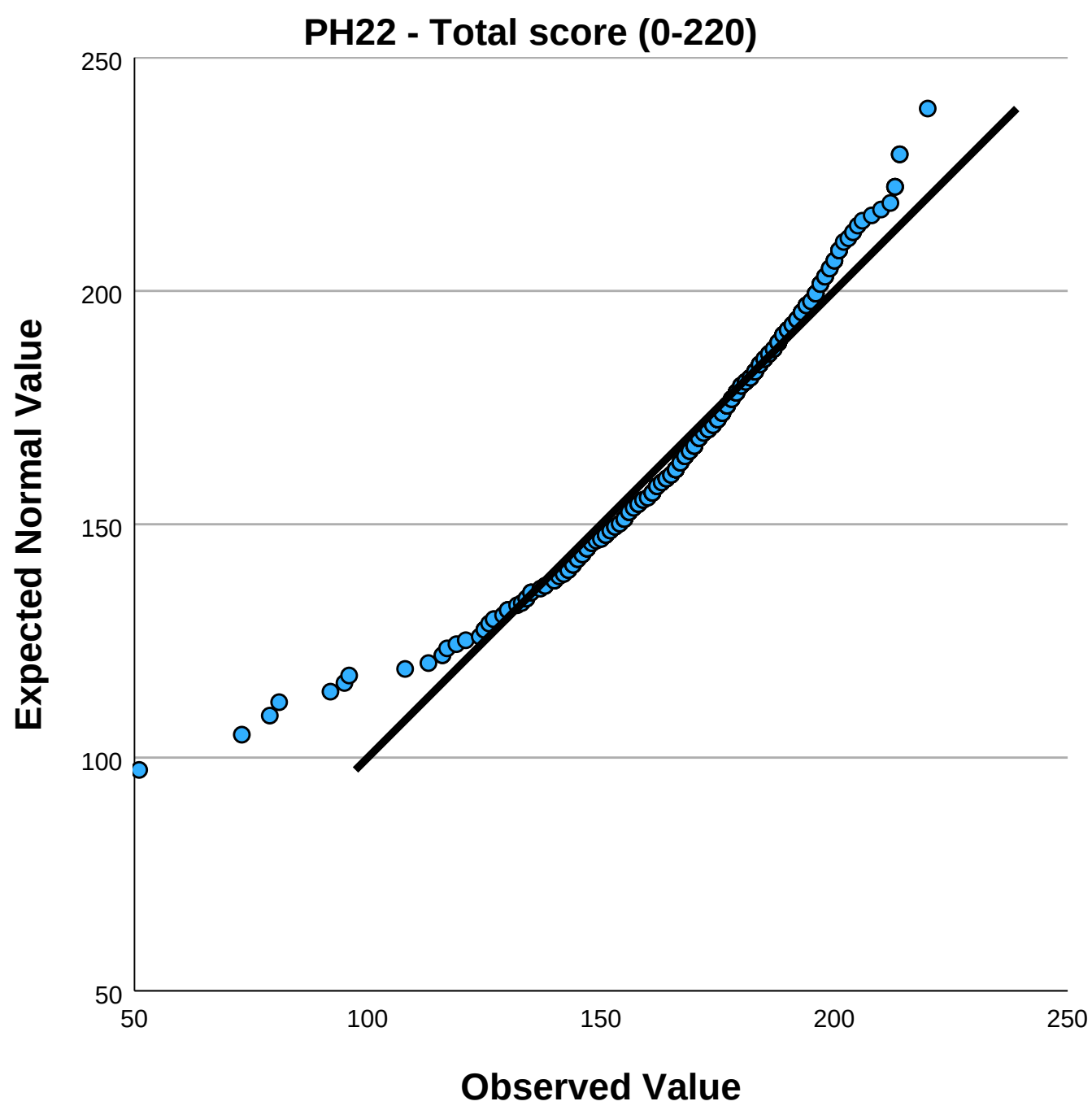
PH22 - Total score (0-220)
Expected Normal Value
Observed Value
250
200
150
100
50
50
100
150
200
250
