# Supplementary material for: The 22-item Positive Health questionnaire (PH22) is reliable and applicable among different (Dutch) hospital populations: A test-retest study design
Source: PLoS One. 2026 Aug 20;21(8):e0356368. doi: 10.1371/journal.pone.0356368 (PMC13492778; doi:10.1371/journal.pone.0356368)
Supplement: Supplementary file 3 [file pone.0356368.s003.pdf]

Histogram

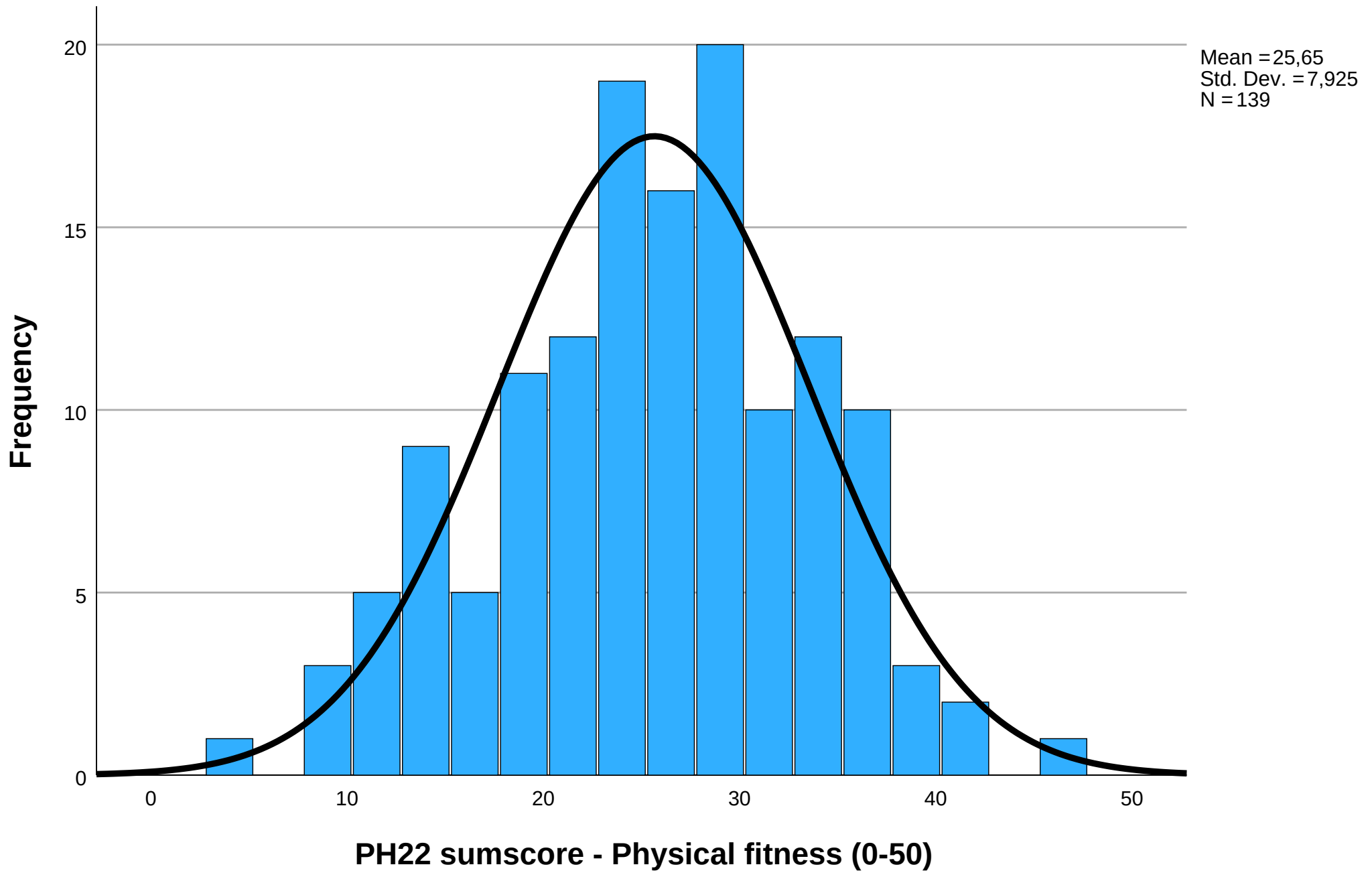
Frequency
20
15
10
5
0
0
10
20
30
40
50
Mean =25,65
Std. Dev. =7,925
N =139
PH22 sumscore - Physical fitness (0-50)

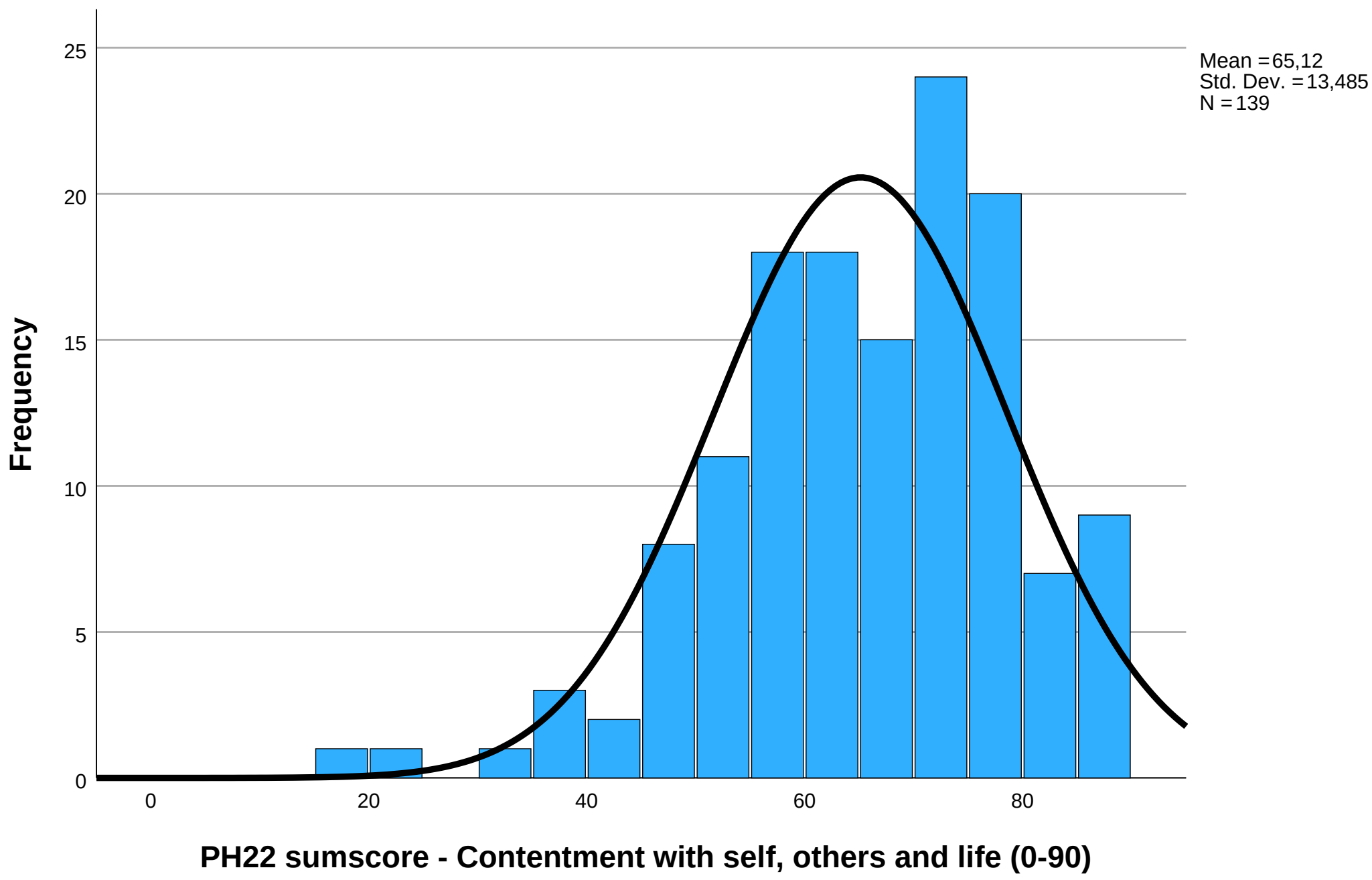
Frequency
25
20
15
10
5
0
0
20
40
60
80
Mean =65,12
Std. Dev. =13,485
N =139
PH22 sumscore - Contentment with self, others and life (0-90)

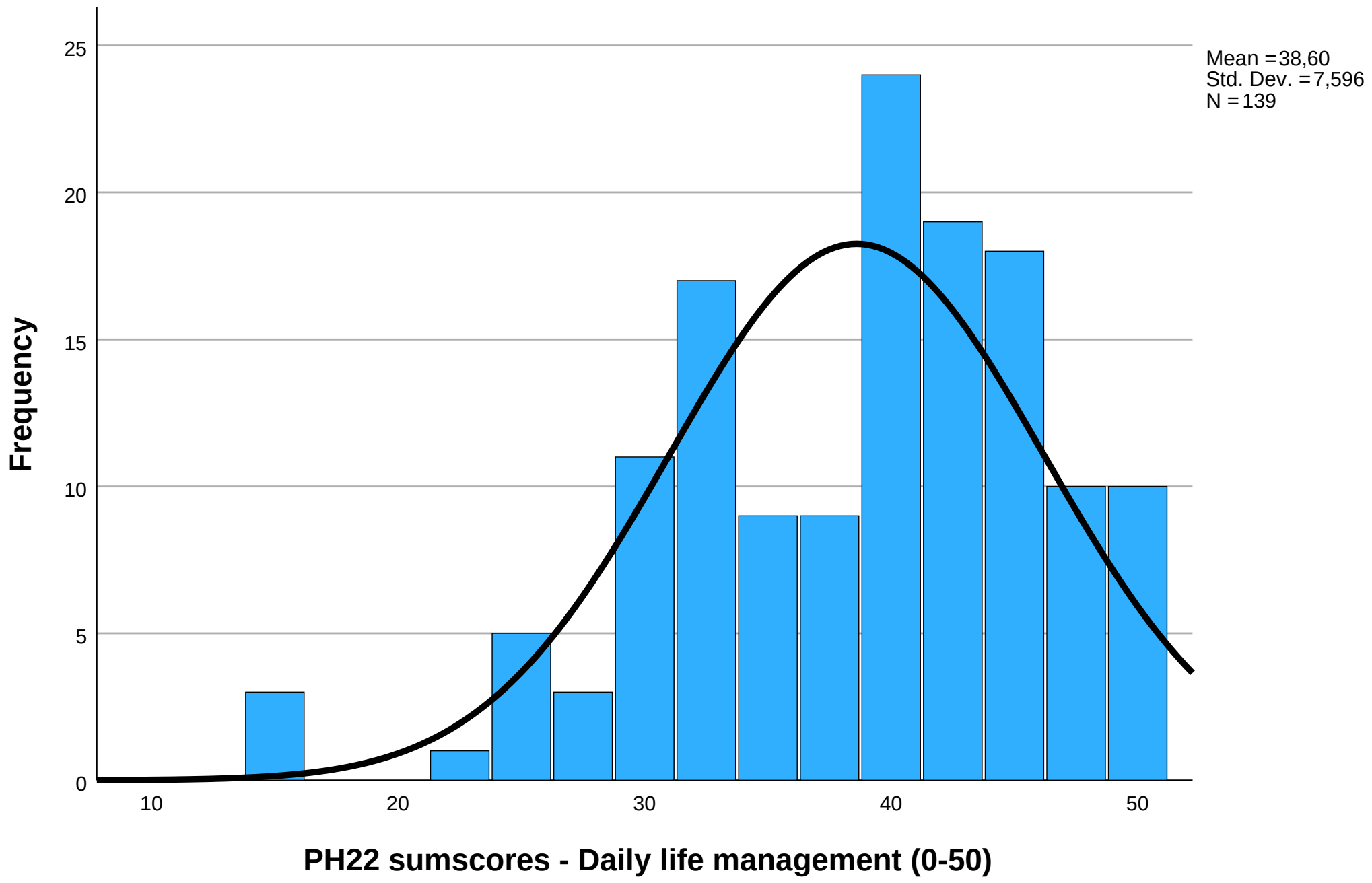
Mean =38,60
Std. Dev. =7,596
N =139
Frequency
0
5
10
15
20
25
10
20
30
40
50
PH22 sumscores - Daily life management (0-50)

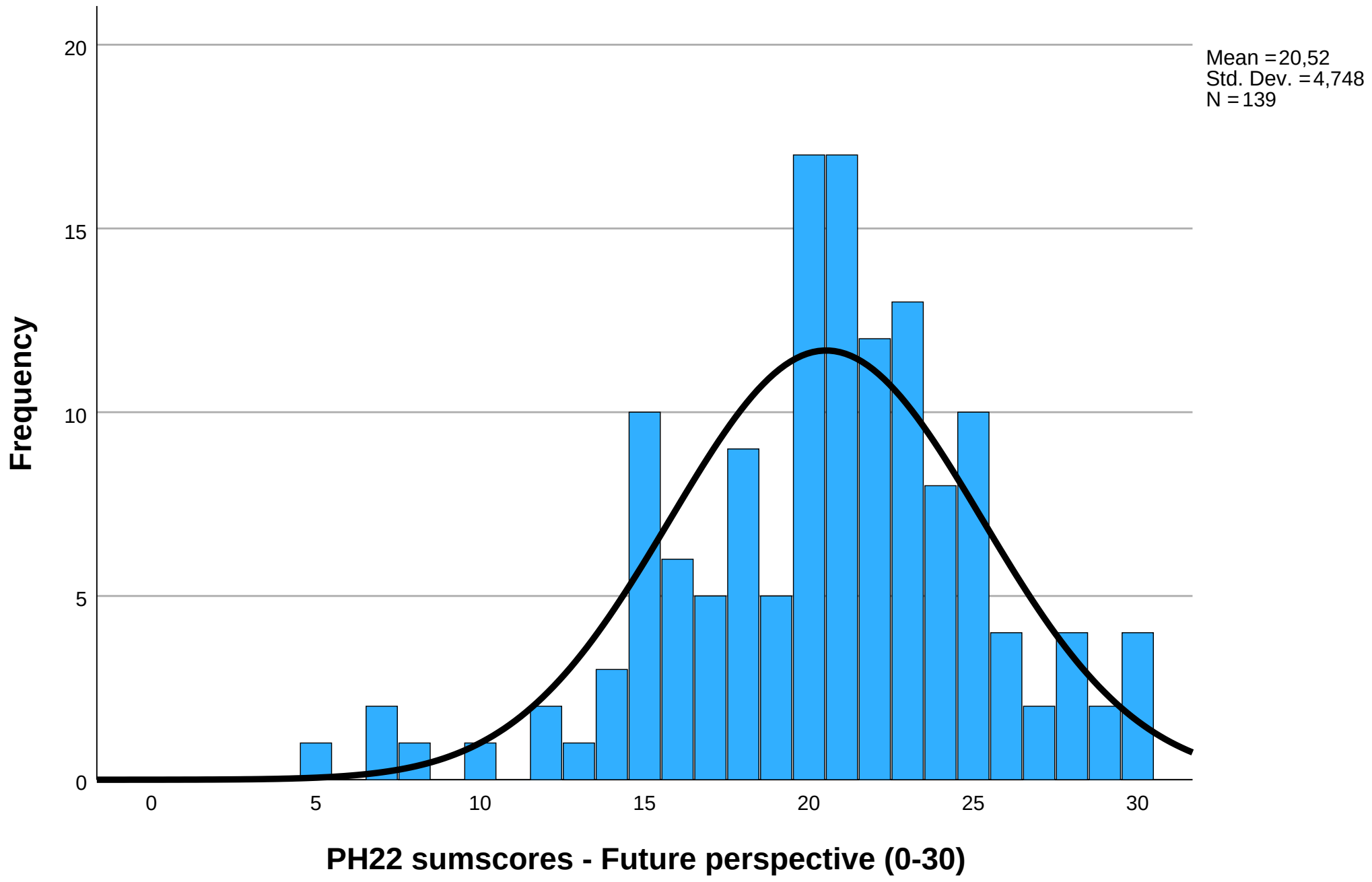
Mean =20,52
Std. Dev. =4,748
N =139
Frequency
0
5
10
15
20
0
5
10
15
20
25
30
PH22 sumscores - Future perspective (0-30)

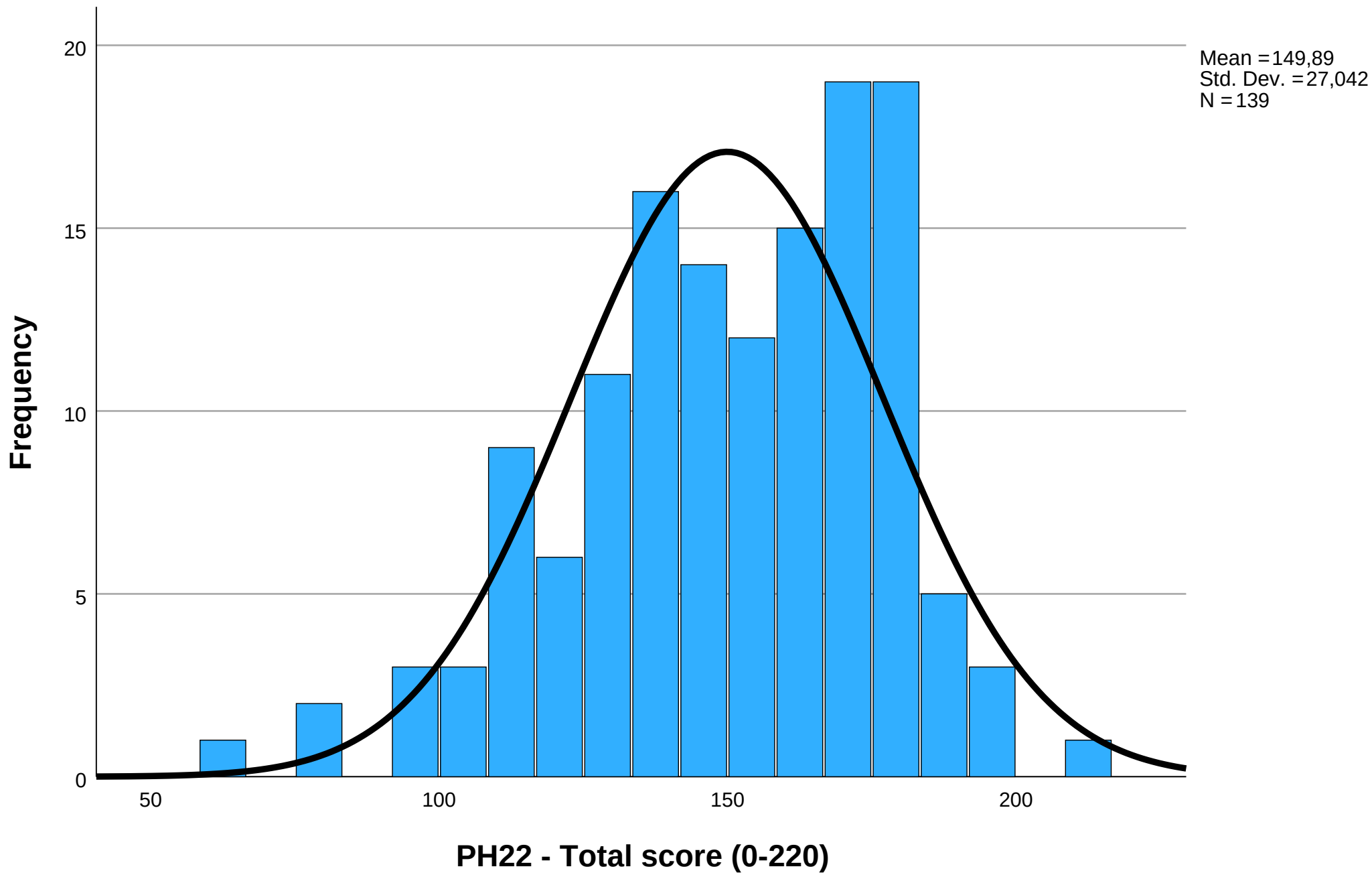

Normal Q-Q Plot

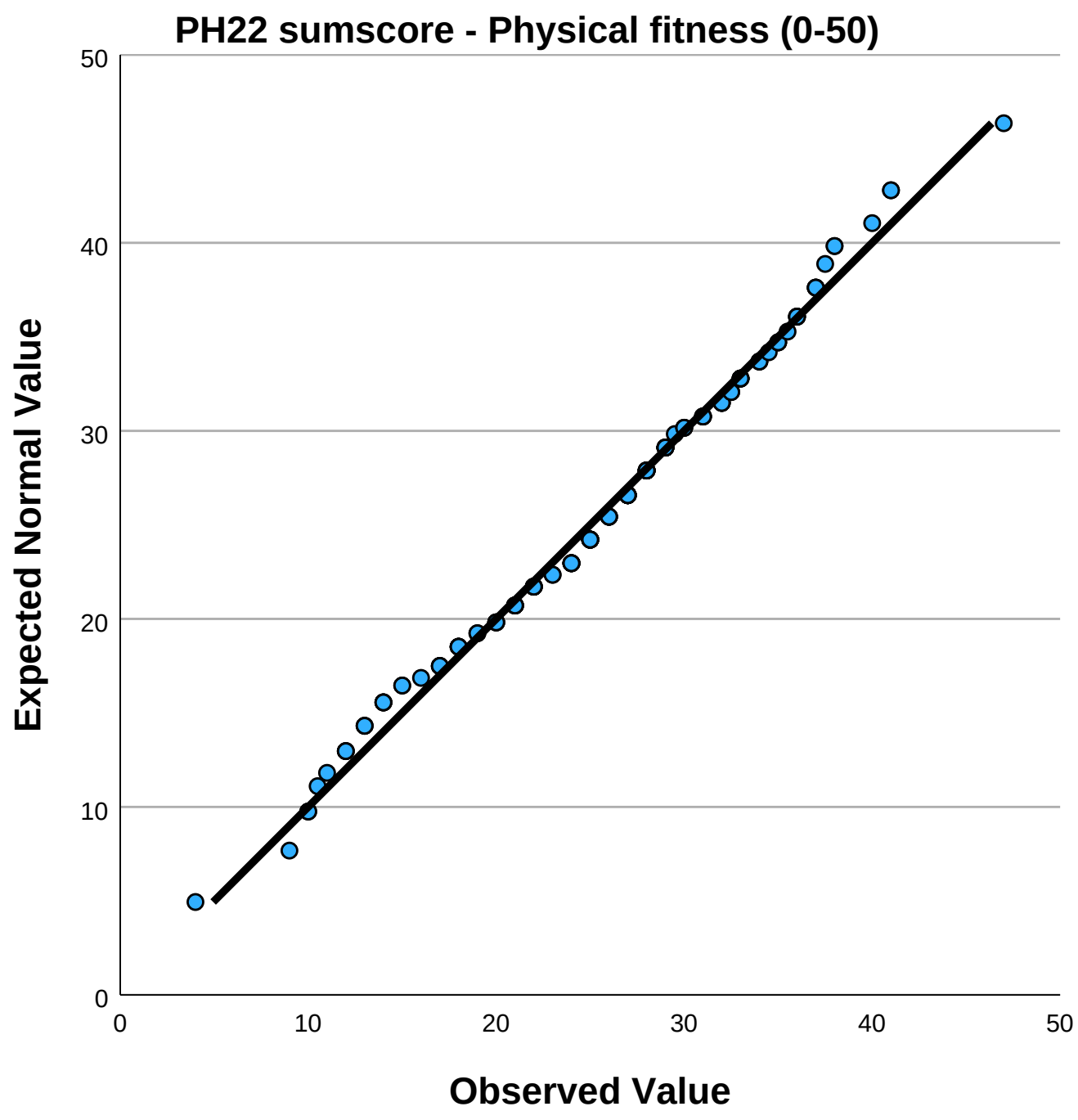

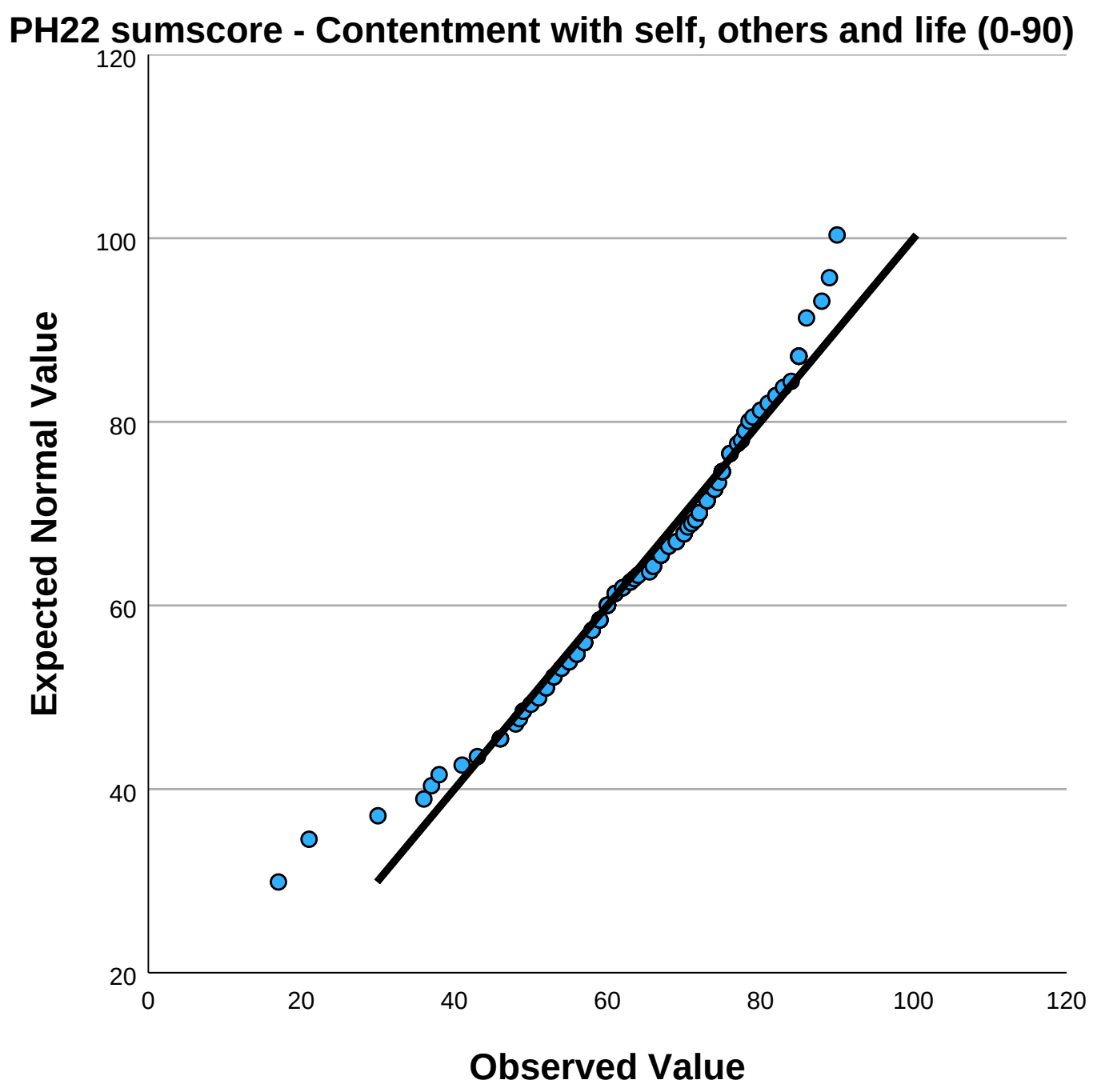
PH22 sumscore - Contentment with self, others and life (0-90)
Expected Normal Value
120
100
80
60
40
20
0
20
40
60
80
100
120
Observed Value

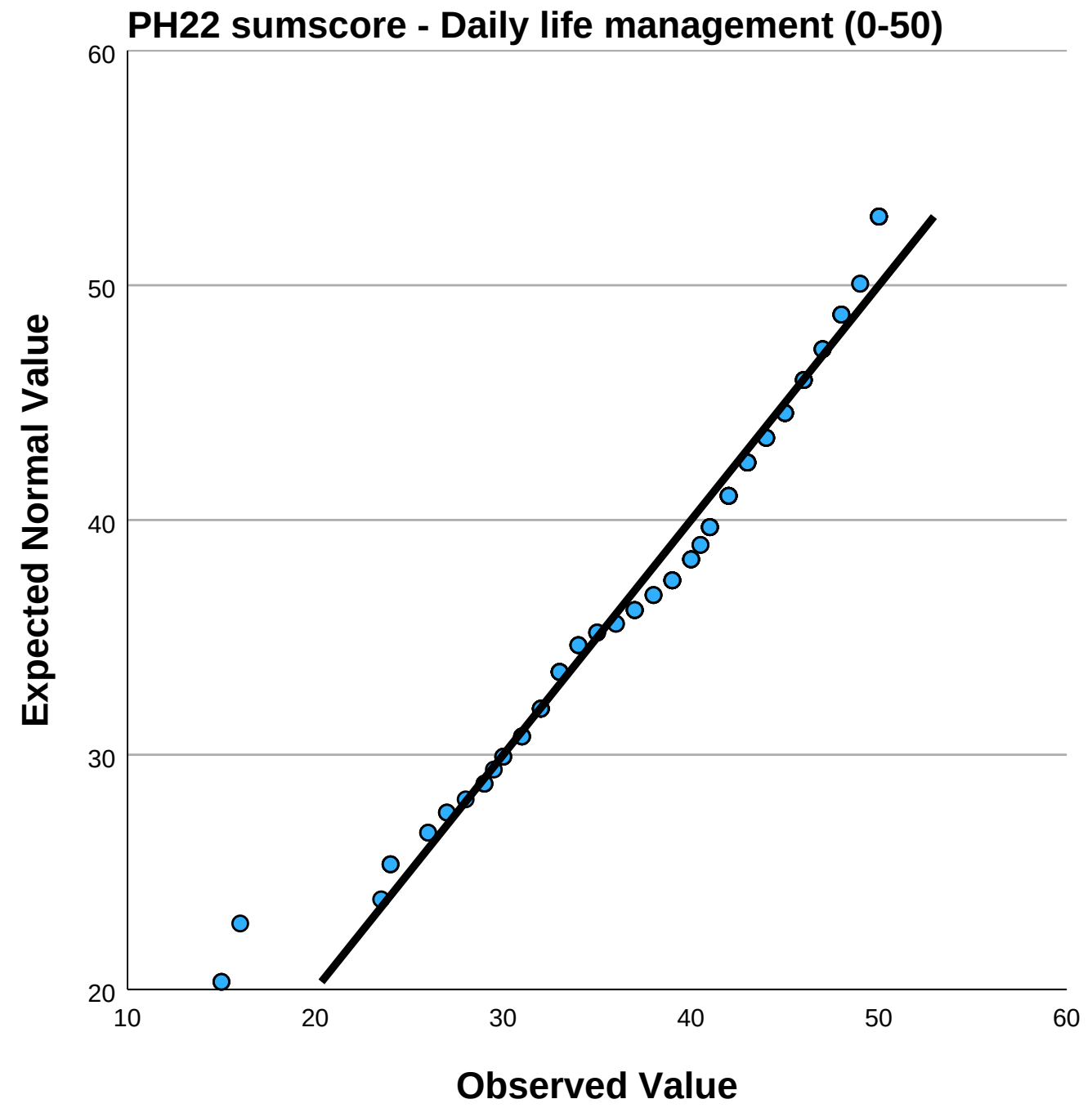
PH22 sumscore - Daily life management (0-50)
Expected Normal Value
60
50
40
30
20
10
20
30
40
50
60
Observed Value

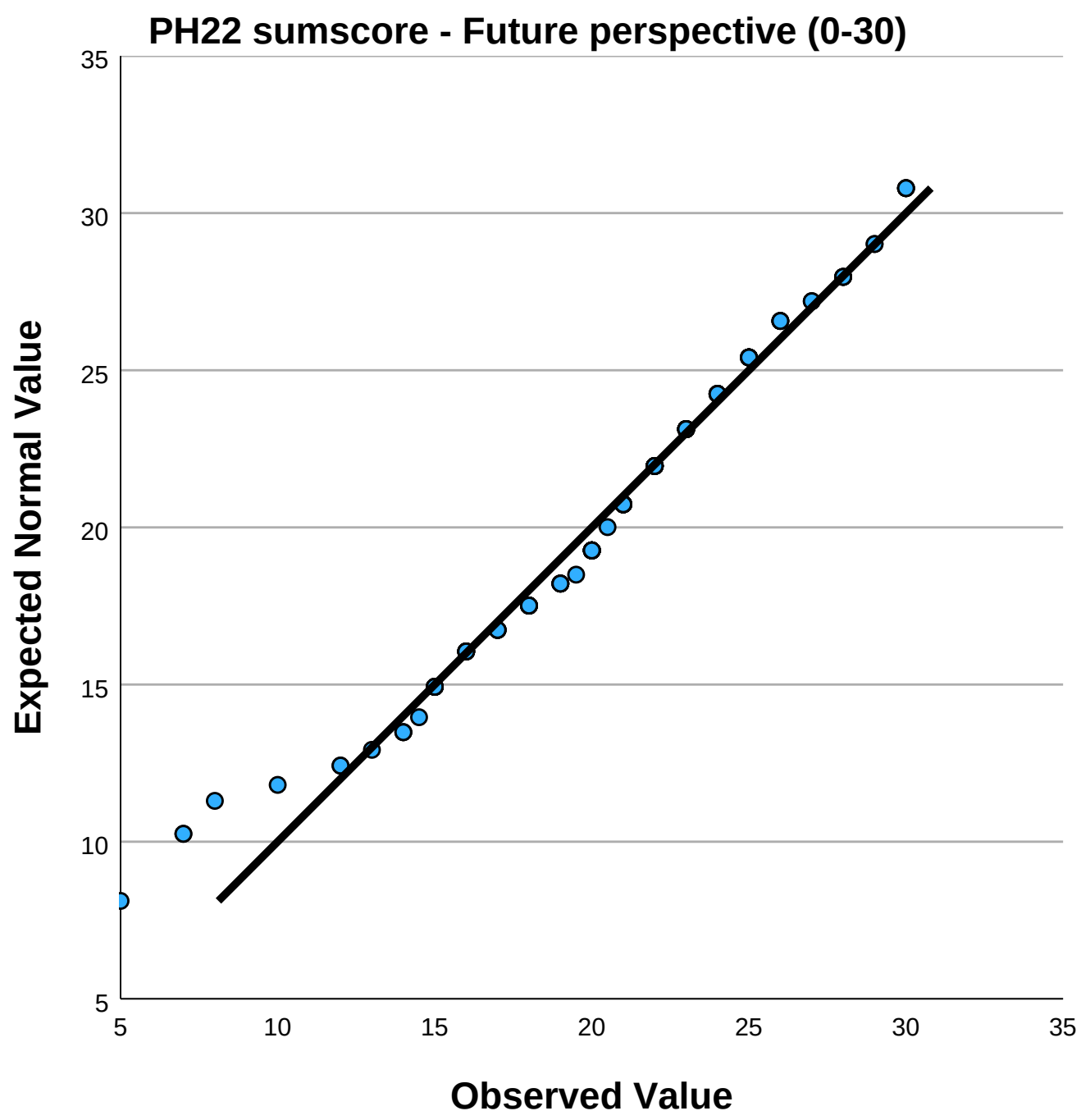
PH22 sumscore - Future perspective (0-30)
Expected Normal Value
Observed Value
5
10
15
20
25
30
35

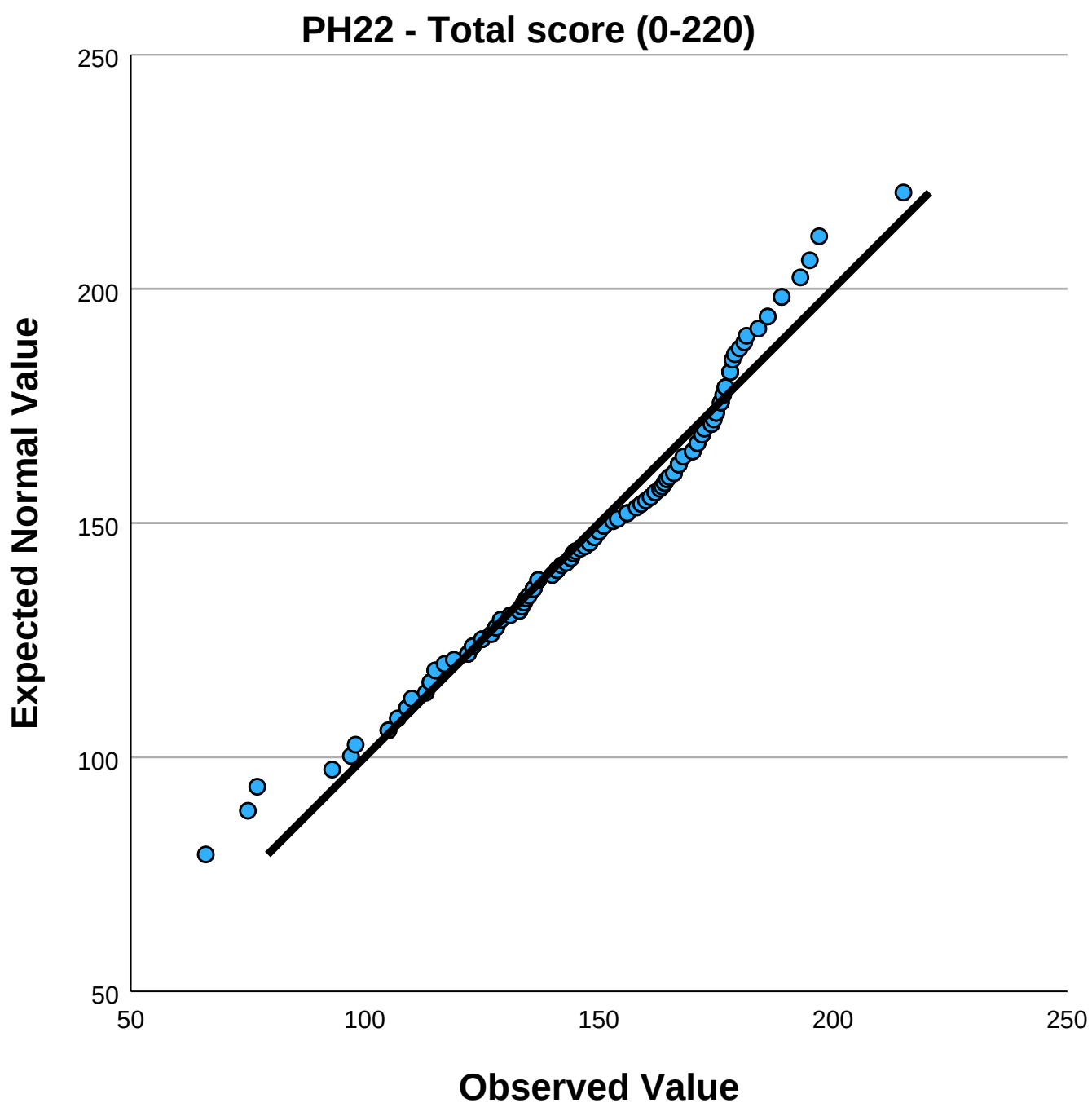
PH22 - Total score (0-220)
Expected Normal Value
Observed Value
50
100
150
200
250
