# Supplementary material for: The 22-item Positive Health questionnaire (PH22) is reliable and applicable among different (Dutch) hospital populations: A test-retest study design
Source: PLoS One. 2026 Aug 20;21(8):e0356368. doi: 10.1371/journal.pone.0356368 (PMC13492778; doi:10.1371/journal.pone.0356368)
Supplement: Supplementary file 4 [file pone.0356368.s005.docx]

**Point-by-point response to the reviewers**

Dear Academic Editor, Maria José Nogueira, Ph.D.,

We thank you and the reviewers for their appraisal of our manuscript and giving us comments for improvement. We have carefully reviewed and discussed the comments and changed the manuscript accordingly. Below, please find the comments of the reviewers, followed by our responses (*Response*) and the changes we made in the manuscript (Changes). We appreciate the opportunity to revise the manuscript. We hope that the revised manuscript will be considered suitable for publication in *PLOS One*. We look forward hearing from you.

Yours sincerely,

Lenny Nahar – van Venrooij

**Editor(s)' Comments**:

Journal Requirements:

When submitting your revision, we need you to address these additional requirements.

*Our response point-by-point:*

1. *The manuscript meets PLOS ONE's style requirements*
2. *We have added additional details regarding how this consent was documented, witnessed, and whether this was approved by the IRB, in the Methods section. See Lines 153, 156-157*

*Ethical approval and consent to participate*

Both studies were conducted in accordance with current public regulations, laws, and the principles of the Declaration of Helsinki.. Informed consent was given by each participant to be included. Informed consent was obtained written for the **general** **hospital study population** and verbal for the **lifestyle outpatient population** (which was documented by their healthcare provider at the electronic patient file). The Medical Ethics Committee of Brabant (Tilburg, the Netherlands) declared that the Medical Research Involving Human Subjects Act (WMO) did not apply to these studies (study numbers NW2024-44, NW2023-10). All procedures including informed consent were approved by our local Research Board and Board of Directors.

3. *Our stated Competing Interest does not alter our adherence to all PLOS ONE policies on sharing data and materials. We included the following statement in our cover letter: "This does not alter our adherence to  PLOS ONE policies on sharing data and materials.”*
4. *We have the rights to share the data as mentioned before in the Data Availability Statement*
5. *Not applicable*
6. *One reference was added to the list in response to the comment of Reviewer 1. Reference 29.* *The reference list is complete and correct.*

Additional Editor Comments:

*Our figures were not created or modified using artificial intelligence (AI).* *Our figures meet the technical requirements (tif,, 300dpi, <10mb).*

**Reviewer 1**

Comments to the Author:
Reviewer #1: No special observations.
There are nowadays some cultural issues, namely related to how people view their health. Some cultural differences can determine perceptions of health.
It seems wise to start including these issues in future work, especially in populations with great cultural diversity.

*Response; Thank you for your important remark. We have added some information to the discussion section to highlight that cultural differences can determine perceptions of health and that cross-cultural validation is advisable. See lines 347-349.*

However, caution should be taken comparing the PH22 scores between more specific groups. It should be noted that also other such as cultural differences can determine perceptions of health (29). Cross-cultural validation is advisable.

**Reviewer 2**
Comments to the Author:
Reviewer #2: Thank you for sharing this excellent article.
Abstract- background should develop the theory further.
In the introduction, they should provide a theoretical context for both positive health and the associated instruments. And in which populations they have been validated.
The methods section states that the second population is overweight. Why was this choice made? Will you validate this instrument for this population? However, the introduction makes no mention of this, so you should provide a justification.
It is also unclear from the introduction which population the instrument is intended to be validated for, which is why I found the methodology a little confusing.

*Response; thank you for your compliment and important comments. We extended the background of the abstract, accordingly. See lines 29-36. We made the text about the theoretical concept of Positive Health more explicit see* *lines 62-68. We added the information about the populations and why the choice for the overweight population visiting the Lifestyle Outpatient Clinic was made. See lines 96, 100-101, and 132-136.*

Lines 29-36

**Abstract**

**Background** There is a growing adoption in (healthcare) organizations of Positive Health, a new theoretical concept of health moving from a disease-oriented perspective toward a more holistic understanding of health. Alongside this, there is a growing need to make the impact of working according to this concept measurable. The validated 22-item Positive Health questionnaire (PH22) has good potential for this purpose. However, information about the test-retest reliability of the PH22 is lacking. Also, its performance in patient populations is unknown.

**Objectives** In addition to the previous validation study in a general population, this study aimed, in line with international guidelines, to evaluate the (test-retest) reliability of the PH22, and its performance among two different hospital populations.

Lines 62-68

In 2011, Huber et al. (4) introduced this dynamic and broad concept of health, defining health as the ability to adapt to physical, emotional, and social challenges in life and to maintain self-management. This new concept of health moved away from a disease-oriented perspective toward a more holistic understanding of health. Subsequently, in 2016, this theoretical concept was further operationalized into a patient-centered concept of health comprising six dimensions: Bodily functions, Mental well-being, Meaningfulness, Quality of life, Social participation, and Daily functioning, called *Positive Health* (PH) (5).

Lines 96, 100-101 (Introduction)

The PH22 was initially developed based on a representative cohort of the general population (11). To meaningfully use PH22 scores for assessing differences between groups and changes over time in order to evaluate the PH approach or patient-centered care, it is essential to quantify the measurement error of the instrument to ensure correct interpretation(13). Moreover, for the application of the PH22 in hospital settings, it is important to expand assessment of its measurement properties for patient populations beyond the general public. Currently, evidence regarding its test-retest reliability or performance of the PH22 in such populations is lacking.

Lines 132-136 (Methods)

The second population was a population with overweight visiting the hospital Lifestyle outpatient clinic, further called the **lifestyle outpatient population**. At the Lifestyle outpatient clinic conversations using the Positive Health approach and its dialogue tool are applied. This outpatient clinic was one of the first departments in this hospital using this Positive Health approach with also potential to be adopted by other hospitals. Therefore, the PH22 was also studied among this specific population. Included were Dutch speaking adult patients (≥18y) with BMI>25 referred to the Lifestyle outpatient clinic by their JBH medical or nursing specialist (see **App. S1** for JBH referral criteria).
