# Supplementary material for: The 22-item Positive Health questionnaire (PH22) is reliable and applicable among different (Dutch) hospital populations: A test-retest study design
Source: PLoS One. 2026 Aug 20;21(8):e0356368. doi: 10.1371/journal.pone.0356368 (PMC13492778; doi:10.1371/journal.pone.0356368)
Supplement: Peer review history

# Peer review: Decision Letter 0

**DOI:** 10.1371/journal.pone.0356368.r001

**Authors:** Maria Nogueira

13 Jul 2026

plosone@plos.org. When you're ready to submit your revision, log on to https://www.editorialmanager.com/pone/ and select the 'Submissions Needing Revision' folder to locate your manuscript file.

- A letter that responds to each point raised by the academic editor and reviewer(s). You should upload this letter as a separate file labeled 'Response to Reviewers'.
- A marked-up copy of your manuscript that highlights changes made to the original version. You should upload this as a separate file labeled 'Revised Manuscript with Track Changes'.
- An unmarked version of your revised paper without tracked changes. You should upload this as a separate file labeled 'Manuscript'.

If you would like to make changes to your financial disclosure, please include your updated statement in your cover letter. Guidelines for resubmitting your figure files are available below the reviewer comments at the end of this letter.

If applicable, we recommend that you deposit your laboratory protocols in protocols.io to enhance the reproducibility of your results. Protocols.io assigns your protocol its own identifier (DOI) so that it can be cited independently in the future. For instructions see: https://journals.plos.org/plosone/s/submission-guidelines#loc-laboratory-protocols. Additionally, PLOS ONE offers an option for publishing peer-reviewed Lab Protocol articles, which describe protocols hosted on protocols.io. Read more information on sharing protocols at https://plos.org/protocols?utm_medium=editorial-email&utm_source=authorletters&utm_campaign=protocols.

As the corresponding author, your ORCID iD is verified in the submission system and will appear in the published article. PLOS supports the use of ORCID, and we encourage all coauthors to register for an ORCID iD and use it as well. Please encourage your coauthors to verify their ORCID iD within the submission system before final acceptance, as unverified ORCID iDs will not appear in the published article. *Only*  the individual author can complete the verification step; PLOS staff *cannot*  verify ORCID iDs on behalf of authors.

We look forward to receiving your revised manuscript.

Kind regards,

Maria José Nogueira, Ph.D.

Academic Editor

PLOS One

Journal Requirements:

When submitting your revision, we need you to address these additional requirements.

1.Please ensure that your manuscript meets PLOS ONE's style requirements, including those for file naming. The PLOS ONE style templates can be found at

https://journals.plos.org/plosone/s/file?id=wjVg/PLOSOne_formatting_sample_main_body.pdf and

https://journals.plos.org/plosone/s/file?id=ba62/PLOSOne_formatting_sample_title_authors_affiliations.pdf

2. In the ethics statement in the Methods, you have specified that verbal consent was obtained. Please provide additional details regarding how this consent was documented and witnessed, and state whether this was approved by the IRB.

3. Thank you for stating the following in the Competing Interests section:

“I have read the journal's policy and the authors of this manuscript have the following competing interests:LNvV and BvdZ co-developed the PH22.”

Please confirm that this does not alter your adherence to all PLOS ONE policies on sharing data and materials, by including the following statement: "This does not alter our adherence to  PLOS ONE policies on sharing data and materials.” (as detailed online in our guide for authors http://journals.plos.org/plosone/s/competing-interests).  If there are restrictions on sharing of data and/or materials, please state these. Please note that we cannot proceed with consideration of your article until this information has been declared.

Please include your updated Competing Interests statement in your cover letter; we will change the online submission form on your behalf.

4. For studies involving third-party data, we encourage authors to share any data specific to their analyses that they can legally distribute. PLOS recognizes, however, that authors may be using third-party data they do not have the rights to share. When third-party data cannot be publicly shared, authors must provide all information necessary for interested researchers to apply to gain access to the data. (https://journals.plos.org/plosone/s/data-availability#loc-acceptable-data-access-restrictions)

For any third-party data that the authors cannot legally distribute, they should include the following information in their Data Availability Statement upon submission:

1) A description of the data set and the third-party source

2) If applicable, verification of permission to use the data set

3) Confirmation of whether the authors received any special privileges in accessing the data that other researchers would not have

4) All necessary contact information others would need to apply to gain access to the data

5. If the reviewer comments include a recommendation to cite specific previously published works, please review and evaluate these publications to determine whether they are relevant and should be cited. There is no requirement to cite these works unless the editor has indicated otherwise.

6. Please review your reference list to ensure that it is complete and correct. If you have cited papers that have been retracted, please include the rationale for doing so in the manuscript text, or remove these references and replace them with relevant current references. Any changes to the reference list should be mentioned in the rebuttal letter that accompanies your revised manuscript. If you need to cite a retracted article, indicate the article’s retracted status in the References list and also include a citation and full reference for the retraction notice.

Additional Editor Comments:

Dear Authors,

Please ensure that you address all of the reviewers' comments. Where a suggested revision cannot be implemented, please provide a clear and well-reasoned justification in your response to the reviewers.

In addition, please include a statement regarding the images used in the manuscript, specifying whether they were created or modified using artificial intelligence (AI). If AI was used, please identify the AI tool employed and indicate its version, in accordance with the journal's requirements.

Thank you for your cooperation.

[Note: HTML markup is below. Please do not edit.]

Reviewers' comments:

Reviewer's Responses to Questions

**Comments to the Author**

1. Is the manuscript technically sound, and do the data support the conclusions?

Reviewer #1: Yes

Reviewer #2: Yes

**********

2. Has the statistical analysis been performed appropriately and rigorously?

Reviewer #1: Yes

Reviewer #2: Yes

**********

3. Have the authors made all data underlying the findings in their manuscript fully available?

The PLOS Data policy

Reviewer #1: Yes

Reviewer #2: Yes

**********

4. Is the manuscript presented in an intelligible fashion and written in standard English?

Reviewer #1: Yes

Reviewer #2: Yes

**********

Reviewer #1: No special observations.

There are nowadays some cultural issues, namely related to how people view their health. Some cultural differences can determine perceptions of health.

It seems wise to start including these issues in future work, especially in populations with great cultural diversity.

Reviewer #2: Thank you for sharing this excellent article.

Abstrat- background should develop the theory further.

In the introduction, they should provide a theoretical context for both positive health and the associated instruments. And in which populations they have been validated.

The methods section states that the second population is overweight. Why was this choice made? Will you validate this instrument for this population? However, the introduction makes no mention of this, so you should provide a justification.

It is also unclear from the introduction which population the instrument is intended to be validated for, which is why I found the methodology a little confusing.

**********

what does this mean?). If published, this will include your full peer review and any attached files.

If you choose “no”, your identity will remain anonymous but your review may still be made public.

**Do you want your identity to be public for this peer review?**  For information about this choice, including consent withdrawal, please see our Privacy Policy

Reviewer #1: **Yes:** Raul Cordeiro

Reviewer #2: **Yes:** Patricia Alves

**********

[NOTE: If reviewer comments were submitted as an attachment file, they will be attached to this email and accessible via the submission site. Please log into your account, locate the manuscript record, and check for the action link "View Attachments". If this link does not appear, there are no attachment files.]

To ensure your figures meet our technical requirements, please review our figure guidelines: https://journals.plos.org/plosone/s/figures

You may also use PLOS’s free figure tool, NAAS, to help you prepare publication quality figures: https://journals.plos.org/plosone/s/figures#loc-tools-for-figure-preparation.

NAAS will assess whether your figures meet our technical requirements by comparing each figure against our figure specifications.

# Author comment: Author response to Decision Letter 1

**DOI:** 10.1371/journal.pone.0356368.r002

20 Jul 2026

Point-by-point response to the reviewers

Dear Academic Editor, Maria José Nogueira, Ph.D.,

We thank you and the reviewers for their appraisal of our manuscript and giving us comments for improvement. We have carefully reviewed and discussed the comments and changed the manuscript accordingly. Below, please find the comments of the reviewers, followed by our responses (Response). The changes we made in the manuscript are added to the uploaded file Response to Reviewers. We appreciate the opportunity to revise the manuscript. We hope that the revised manuscript will be considered suitable for publication in PLOS One. We look forward hearing from you.

Yours sincerely,

Lenny Nahar – van Venrooij

Editor(s)' Comments:

Journal Requirements:

When submitting your revision, we need you to address these additional requirements.

Our response point-by-point:

1. The manuscript meets PLOS ONE's style requirements

2. We have added additional details regarding how this consent was documented, witnessed, and whether this was approved by the IRB, in the Methods section. See Lines 153, 156-157

3. Our stated Competing Interest does not alter our adherence to all PLOS ONE policies on sharing data and materials. We included the following statement in our cover letter: "This does not alter our adherence to PLOS ONE policies on sharing data and materials.”

4. We have the rights to share the data as mentioned before in the Data Availability Statement

5. Not applicable

6. One reference was added to the list in response to the comment of Reviewer 1. Reference 29. The reference list is complete and correct.

Additional Editor Comments:

Our figures were not created or modified using artificial intelligence (AI). Our figures meet the technical requirements (tif,, 300dpi, <10mb).

Reviewer 1

Comments to the Author:

Reviewer #1: No special observations.

There are nowadays some cultural issues, namely related to how people view their health. Some cultural differences can determine perceptions of health.

It seems wise to start including these issues in future work, especially in populations with great cultural diversity.

Response; Thank you for your important remark. We have added some information to the discussion section to highlight that cultural differences can determine perceptions of health and that cross-cultural validation is advisable. See lines 347-349.

Reviewer 2

Comments to the Author:

Reviewer #2: Thank you for sharing this excellent article.

Abstract- background should develop the theory further.

In the introduction, they should provide a theoretical context for both positive health and the associated instruments. And in which populations they have been validated.

The methods section states that the second population is overweight. Why was this choice made? Will you validate this instrument for this population? However, the introduction makes no mention of this, so you should provide a justification.

It is also unclear from the introduction which population the instrument is intended to be validated for, which is why I found the methodology a little confusing.

Response; thank you for your compliment and important comments. We extended the background of the abstract, accordingly. See lines 29-36. We made the text about the theoretical concept of Positive Health more explicit see lines 62-68. We added the information about the populations and why the choice for the overweight population visiting the Lifestyle Outpatient Clinic was made. See lines 96, 100-101, and 132-136.

Attachment Submitted filename: Response to reviewers PLOS One_17 July 2026.docx

# Peer review: Decision Letter 1

**DOI:** 10.1371/journal.pone.0356368.r003

**Authors:** Maria Nogueira

3 Aug 2026

The 22-item Positive Health questionnaire (PH22) is reliable and applicable among different (Dutch) hospital populations: A test-retest study design

PONE-D-26-25023R1

Dear Dr. Lenny M.W. Nahar - van Venrooij

We’re pleased to inform you that your manuscript has been judged scientifically suitable for publication and will be formally accepted for publication once it meets all outstanding technical requirements.

Within one week, you’ll receive an e-mail detailing the required amendments. When these have been addressed, you’ll receive a formal acceptance letter and your manuscript will be scheduled for publication.

An invoice will be generated when your article is formally accepted. Please note, if your institution has a publishing partnership with PLOS and your article meets the relevant criteria, all or part of your publication costs will be covered. Please make sure your user information is up-to-date by logging into Editorial Manager at Editorial Manager® and clicking the ‘Update My Information' link at the top of the page. For questions related to billing, please contact billing support.

If your institution or institutions have a press office, please notify them about your upcoming paper to help maximize its impact. If they’ll be preparing press materials, please inform our press team as soon as possible -- no later than 48 hours after receiving the formal acceptance. Your manuscript will remain under strict press embargo until 2 pm Eastern Time on the date of publication. For more information, please contact onepress@plos.org.

Kind regards,

Maria José Nogueira, Ph.D.

Academic Editor

PLOS One

Additional Editor Comments (optional):

The authors have satisfactorily addressed the previous recommendations, and the manuscript has improved substantially.

In its current form, it is suitable for publication.

Reviewers' comments:

Reviewer's Responses to Questions

**Comments to the Author**

Reviewer #2: All comments have been addressed

**********

2. Is the manuscript technically sound, and do the data support the conclusions?

Reviewer #2: Yes

**********

3. Has the statistical analysis been performed appropriately and rigorously?

Reviewer #2: Yes

**********

4. Have the authors made all data underlying the findings in their manuscript fully available?

The PLOS Data policy

Reviewer #2: Yes

**********

5. Is the manuscript presented in an intelligible fashion and written in standard English?

Reviewer #2: Yes

**********

Reviewer #2: Congratulations on the review, The review made perfect sense and is therefore now ready for publication

**********

what does this mean?). If published, this will include your full peer review and any attached files.

If you choose “no”, your identity will remain anonymous but your review may still be made public.

**Do you want your identity to be public for this peer review?**  For information about this choice, including consent withdrawal, please see our Privacy Policy

Reviewer #2: **Yes:** Patricia Alves

**********

# Editor report: Acceptance letter

**DOI:** 10.1371/journal.pone.0356368.r004

**Authors:** Maria Nogueira

PONE-D-26-25023R1

PLOS One

Dear Dr. Nahar - van Venrooij,

I'm pleased to inform you that your manuscript has been deemed suitable for publication in PLOS One. Congratulations! Your manuscript is now being handed over to our production team.

At this stage, our production department will prepare your paper for publication. This includes ensuring the following:

* All references, tables, and figures are properly cited

* All relevant supporting information is included in the manuscript submission,

* There are no issues that prevent the paper from being properly typeset

You will receive further instructions from the production team, including instructions on how to review your proof when it is ready. Please keep in mind that we are working through a large volume of accepted articles, so please give us a few days to review your paper and let you know the next and final steps.

Lastly, if your institution or institutions have a press office, please let them know about your upcoming paper now to help maximize its impact. If they'll be preparing press materials, please inform our press team within the next 48 hours. Your manuscript will remain under strict press embargo until 2 pm Eastern Time on the date of publication. For more information, please contact onepress@plos.org.

You will receive an invoice from PLOS for your publication fee after your manuscript has reached the completed accept phase. If you receive an email requesting payment before acceptance or for any other service, this may be a phishing scheme. Learn how to identify phishing emails and protect your accounts at https://explore.plos.org/phishing.

If we can help with anything else, please email us at customercare@plos.org.

Thank you for submitting your work to PLOS ONE and supporting open access.

Kind regards,

PLOS ONE Editorial Office Staff

on behalf of

Professor Maria José Nogueira

Academic Editor

PLOS One
